# Interfacial Stress Regulates Plasticity and Drug Resistance at the Breast Cancer‐Host Interface

**DOI:** 10.1002/advs.202509361

**Published:** 2025-06-26

**Authors:** Bram G. Soliman, Peilin Tian, Jiuyu Cui, Kristopher A. Kilian, J. Justin Gooding

**Affiliations:** ^1^ School of Chemistry University of New South Wales Sydney New South Wales 2052 Australia; ^2^ Australian Centre for NanoMedicine University of New South Wales Sydney New South Wales 2052 Australia; ^3^ School of Materials Science and Engineering University of New South Wales Sydney New South Wales 2052 Australia

**Keywords:** cancer, cellular heterogeneity, confinement, drop‐on‐demand printing, drug delivery, drug resistance, solid stress

## Abstract

The confinement of breast cancer cells at the interface of the mammary gland lumen and its surrounding extracellular matrix is thought to be a key physical driver of cellular plasticity. The relationship between confinement‐induced solid stress and drug resistance is not well understood due to a scarcity of models that faithfully isolate the contribution of confinement on cancer cell behavior. Herein, drop‐on‐demand printing is used to uniquely replicate the spatial compartmentalization of the native cancer‐host interface: MCF‐7 breast cancer cells are dispensed within bioinert cup‐shaped alginate‐based hydrogels in high‐throughput to yield “confined” spheroids. Hydrogel confinement affects the emergence of CD44+‐CD133+ cells at the spheroid interface that drives a two‐fold increase in doxorubicin/tamoxifen resistance compared to control “unconfined” spheroids. Whilst the peripheral drug‐resistant phenotype is observed clinically, the model is unique in facilitating the emergence of this population in an in vitro setting. Pharmacological modulation of mechanotransduction proteins (YAP, myosin), abrogated the emergence of this peripheral phenotype, implicating mechanotransduction pathways as an effective way to target solid stress‐induced drug resistance. Together, this supports an “interfacial stress—stemness—drug resistance” relationship that sheds new light on the existing paradigm of spatial emergence of drug resistance in breast cancer.

## Graphical abstract

Drop‐on‐demand printing is used to replicate the spatial compartmentalization of the breast cancer‐host interface. Hydrogel confinement affects phenotypic changes in cells at the spheroid interface that drives a two‐fold increase in chemotherapeutic resistance compared to control unconfined spheroids. This supports an “interfacial stress—stemness—drug resistance” relationship that sheds light on how drug‐resistant niches spatially emerge in breast cancer.

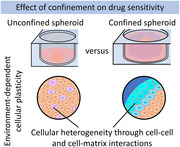

## 1 Introduction

Breast cancer remains the cancer with the highest incidence amongst women,^[^ ^1^ ^]^ ranking as the 5th highest cause of cancer‐related deaths.^[^ ^2^ ^]^ Early diagnosis tools such as mammography screening and the development of targeted adjuvant therapies have yielded improvements in patient prognosis, preventing progression to invasive cancers in >70% of cases.^[^ ^1^ ^]^ Non‐responsiveness and partial responsiveness to drugs through the development of drug resistance however is thought to be the main driver of treatment failure in remaining cases, allowing tumor progression to non‐curable metastatic breast cancer.^[^ ^3^ ^]^ An improved understanding of the processes underlying breast cancer drug sensitivity is thus crucial in defining strategies to overcome therapeutic resistance.

The vast majority of breast cancers originate from epithelial cells lining the mammary duct wall.^[^ ^4^ ^]^ Understanding the interface between these breast cancers, termed ductal carcinomas, and the surrounding breast tumor microenvironment is critical to mapping the factors that influence cell state plasticity and the development of drug resistance. During cancer progression, tensile stresses are generated at this interface due to the expansion of the tumor mass and confinement dictated by fibrotic breast tissue.^[^ ^5^ ^]^ At the same time, compressive stress from the host tissue leads to the accumulation of mechanical stress within the tumor^[^ ^6^, ^7^, ^8^ ^]^ and transcriptional changes at the cellular level. Solid stress‐induced transcriptional changes have been implicated in the metastatic cascade, inducing invasion through activating epithelial‐to‐mesenchymal transition.^[^ ^9^ ^]^ Solid stress also modulates intracellular tension^[^ ^10^ ^]^ and nuclear compliance,^[^ ^11^ ^]^ increasing cancer cell malleability to enable invasion into the host tissue.

Evidence relating solid stress to chemoresistance is much less abundant and mainly indirect. Arrested tumor growth due to matrix confinement for instance leads to the emergence of low‐proliferative cancer cells within the tumor core that are less susceptible to chemotherapeutic agents that target proliferative pathways.^[^ ^5^, ^12^, ^13^, ^14^ ^]^ Furthermore, desmoplastic breast tissue environments constitute a physical barrier for drug access.^[^ ^15^ ^]^ Confinement of blood vessels can also trigger hypoxia‐induced signaling, which in turn can drive the emergence of drug‐resistant cells within the tumor core.^[^ ^16^, ^17^ ^]^ However, whether solid stress can directly contribute to chemoresistance is not well understood.^[^ ^6^, ^18^ ^]^ Our group has demonstrated within 2D models that interfacial stresses can lead to an upregulation in drug efflux pump and drug resistance marker expression, suggesting a role for the stress‐driven emergence of drug‐resistant populations.^[^ ^19^, ^20^ ^]^ Consequently, further investigation into the relationship between solid stress induced through matrix‐induced confinement and drug resistance within the context of a biomimetic 3D breast cancer model is warranted.

Classical in vitro spheroid culture methods such as the cultivation of cancer cells within low‐adherent plates generate a direct interface between the cancer spheroid and culture media with minimal exertion of solid stress at this interface. Alternatively, spheroids can be embedded within a hydrogel, a highly hydrated network composed of hydrophilic polymers, that are widely used as mimics of the tissue microenvironment.^[^ ^21^, ^22^ ^]^ This approach allows the investigation of spheroid response to solid stress from the confining hydrogel in a hydrogel stiffness‐dependent manner. This body of work has however resulted in conflicting outcomes; the exertion of solid stress has been correlated to an attenuation in breast cancer spheroid growth,^[^ ^23^ ^]^ which in turn has been linked to insensitivity to chemotherapeutics due to the low‐proliferative cell state of these spheroids.^[^ ^24^, ^25^ ^]^ Contrastingly, other studies found that solid stress induced chemotherapeutic resistance through increased proliferation in a stiffness‐^[^ ^26^ ^]^ and viscoelasticity‐dependent^[^ ^27^ ^]^ manner. Whilst these models are valuable tools to probe the mechanistic roles of solid stress, the models do not replicate the compartmentalization of cancer within a lumen space in the confinement of an extracellular matrix‐mimic.

**Figure 1:**
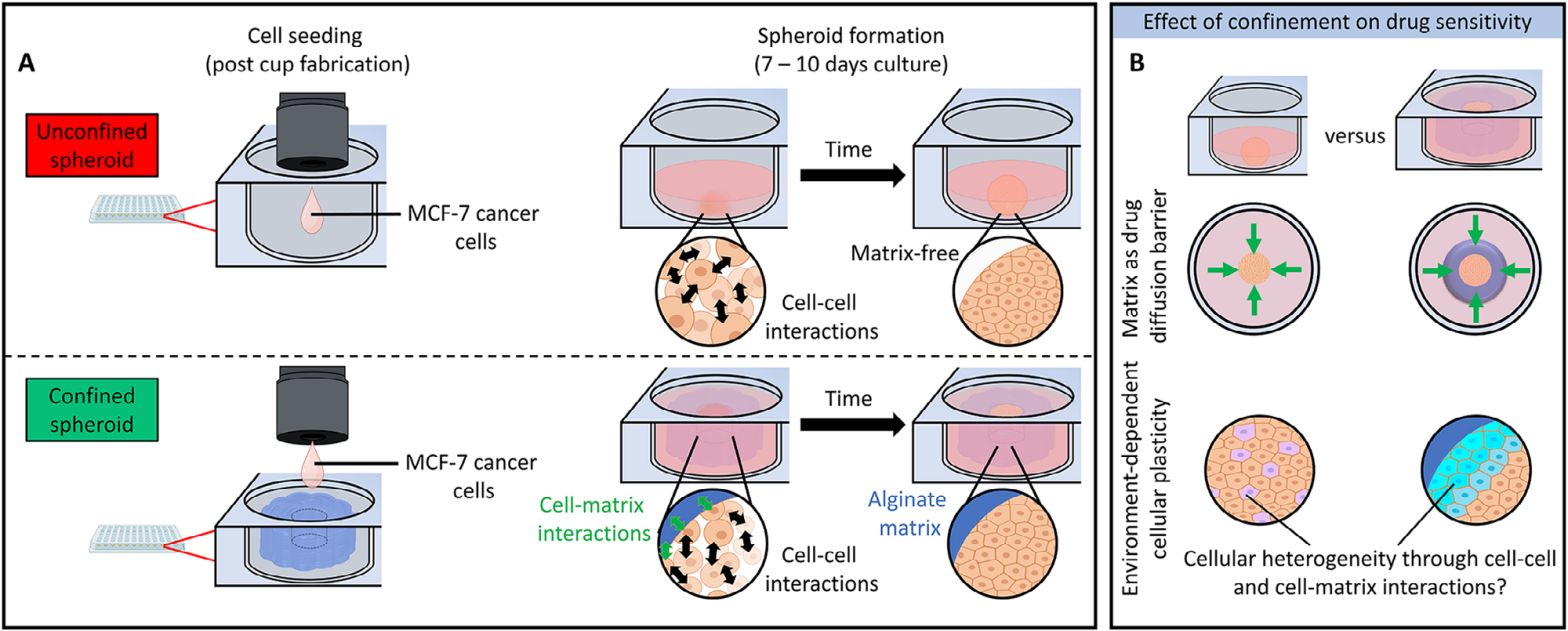
Studying the effect of confinement on drug resistance in breast cancer as enabled through drop‐on‐demand printing. A) MCF‐7 cell suspensions were dispensed directly within low adherent culture plates wherein spheroid formation occurs over time through cell‐cell interactions. These spheroids directly interface with the surrounding media, lacking physical confinement, and were thus termed unconfined spheroids. Confined spheroids were generated by dispensing MCF‐7 cell suspensions within the cavities of printed cup structures. Spheroids formed within the confines of the cup cavities were directly surrounded by the cup cavities and were thus hypothesized to experience physical confinement. B) Direct comparison between unconfined and confined spheroids of similar shape and size was hypothesized to enable a study into the effect of confinement on matrix‐dependent drug diffusion and confinement‐dependent emergence of drug resistance to cancer cell plasticity. The figure contains elements created with BioRender.com.

Here, we explored drop‐on‐demand printing^[^ ^28^, ^29^, ^30^, ^31^ ^]^ to effectively replicate the spatial compartmentalization of the native cancer‐host tissue interface. We hypothesized that mimicry of the native anatomy of the mammary duct would capture cancer heterogeneity and enable probing of the effects of interfacial solid stress on drug resistance in breast cancer. Our approach involved the fabrication of cup‐shaped alginate hydrogels through sequential dispensing of calcium chloride and alginate droplets in a droplet‐by‐droplet, layer‐by‐layer fashion (Figure S1, Supporting Information). The dispensing of a high‐density suspension of non‐invasive MCF‐7 breast cancer cells within the cavities of these cups—and subsequent cultivation of these cells into breast cancer spheroids—served to mimic the “tumor tissue‐physical microenvironment” observed in native ductal carcinomas at early stages preceding dissemination. Comparative analysis between this model, wherein the hydrogel physically confined cancer spheroids (referred to as confined spheroids), and unconfined cancer spheroids cultivated within low‐adherent plates (referred to as unconfined spheroids) enabled decoupling of the effect of confinement‐induced forces such as solid stress on downstream signal transduction (**Figure** **1**A). Chemotherapeutic access/diffusivity and the emergence of cellular heterogeneity were investigated as key contributors to breast cancer drug resistance (Figure 1B). Through investigating the dependency of these processes on the nature of the cancer‐host interface, a direct link between physical confinement and the emergence of cellular heterogeneity at the cancer‐host interface was uncovered that expands on the current breast cancer drug resistance paradigm.

## 2 Results

### 2.1 Shape and Size of Spheroids Could Be Controlled Independent of Interfacial Confinement

We hypothesized that the effect of spheroid confinement on drug resistance would be possible to investigate through a direct comparison between spheroids confined within our drop‐on‐demand printed alginate cups (confined spheroids), with a counterpart that lacks confinement and was rather grown in a low‐adherent culture plate (unconfined spheroids). To establish a fair comparison between these experimental groups, it was imperative that the unconfined and confined spheroids were similar in shape and size. In this way, a comparison was possible wherein differences in drug resistance between unconfined and confined spheroids were purely dependent on the presence of a confining hydrogel.

**Figure 2:**
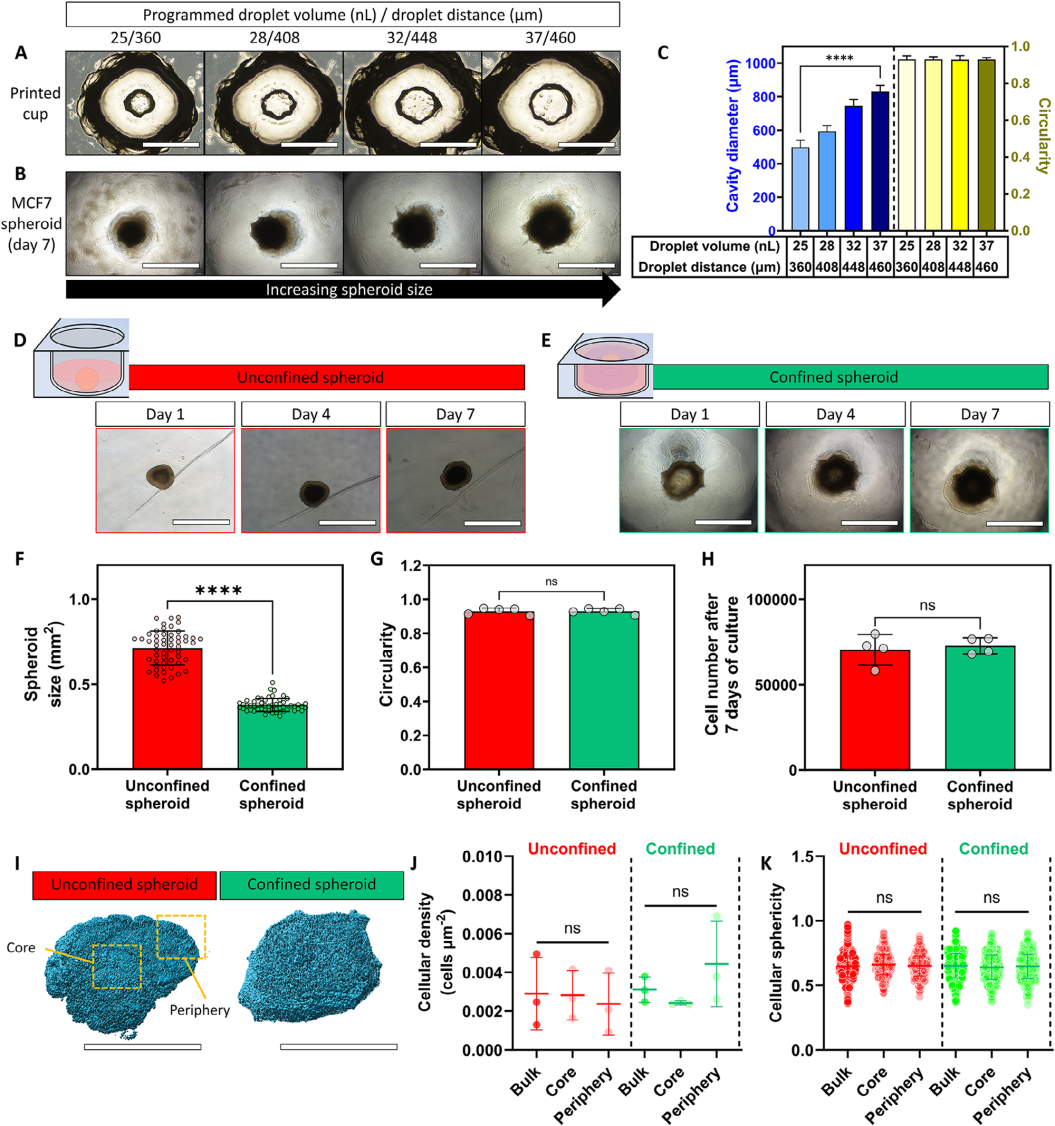
MCF‐7 spheroid growth in unconfined and confined conditions. A–C) Drop‐on‐demand printing of alginate‐based hydrogel cups with tailorable cup cavity sizes (A), wherein MCF‐7 cells aggregated to conform to cavity size and shape after a culture period of seven days (B). Quantitative analysis of cup cavity diameter and circularity (C) demonstrated that cavity size could be tailored independently of cavity shape. D) MCF‐7 cell aggregation toward the formation of spheroids within a well of a low‐adherent U‐shaped culture plate. E) MCF‐7 cell aggregation toward the formation of spheroids within the confined of printed alginate‐based hydrogel cups. F–H) Comparison between unconfined and confined spheroids in spheroid size across 50 individual spheroids (F), spheroid circularity (G), and cell number (H). I,J) Surface‐rendered images of nuclear staining of spheroids were generated in Imaris (I) for quantification of cellular density (J) and cellular sphericity as a measure of cellular shape K). Scale bars = 1 mm (A, B, D, and E) and 500 µm (I). Independent sample t‐test between experimental groups. Statistical non‐significance was indicated with (ns = not significant). Graphs show mean ± SD. N = 3.

Cups with circular cavities could be reproducibly fabricated in high throughput, within a short timeframe (<90 min for a full 96‐well plate). Printing parameters could be adjusted to tailor the cavity size whilst maintaining the circular shape (cup cavity diameter ranging from 498 ± 42 µm to 831 ± 36 µm whilst circularity was maintained at 0.93, **Figure** **2**A–C). Cavity size and shape control were achieved by adjusting the droplet volume and distance between two neighboring droplets (see Figure S2, Supporting Information for further detail). The printing process was cytocompatible as evidenced by high (>90%) Michigan Cancer Foundation‐7 (MCF7) cell viability after dispensing the cells within the cup cavities (Figure S3, Supporting Information). Confinement did not affect cellular viability over the seven‐day culture period, and cells remained metabolically active during this period. Moreover, the initial MCF7 cell number present within the cup cavity was comparable to the unconfined spheroids (Figure S3, Supporting Information). Regardless of cup cavity size, MCF‐7 cells conformed to the cavity shape and size over a culture period of seven days (Figure 2B), thereby allowing a means to tailor spheroid size through adjusting the cup cavity design. In this way, the spheroid size was successfully and reproducibly tailored in the range of 0.27 ± 0.06 mm^2^ and 0.86 ± 0.06 mm^2^ (Figure S4A, Supporting Information). Cups with a cavity diameter of 745 ± 39 µm, yielding MCF‐7 spheroids with a size of 0.56 ± 0.06 mm^2^, were adopted for the rest of this work as these spheroids closely approximated the size of an unconfined spheroid (0.50 ± 0.04 mm^2^) as determined from Brightfield imaging. The unconfined and confined spheroid volumes, as estimated from the Brightfield images, were also found to be similar between both groups (Figure S5, Supporting Information). It was noted that the growth kinetics of unconfined and confined spheroids were dissimilar (Figure 2D,E); in unconfined conditions, MCF‐7 cells formed a spheroid within a day, which then increased in size during the culture period. In confined conditions, MCF‐7 cells aggregated toward the periphery of the cavities to form a ring‐like spheroid, which then grew inward to fill most of the cavity of the cup within four days with the cup cavity size remaining constant (Figure S6, Supporting Information). Regardless, no significant differences were observed in spheroid size (Figure 2F), shape (circularity: 0.91 ± 0.02 and 0.92 ± 0.03, respectively, *p* = 0.9729, Figure 2G), and total cell number (4.2 ± 0.7 × 10^4^ and 5.3 ± 0.6 × 10^4^ cells per spheroid, respectively, *p* = 0.2391, Figure 2H) after five days of culture. No significant differences were observed in the “bulk” (i.e., averaged from all the cells within each spheroid) cellular density and morphology within the unconfined and confined conditions (Figure 2I–K). This observation aligned with previous work from our group where we found similar cellular densities between free‐flowing, unconfined, neuroblastoma spheroids and spheroids grown within the cavities of the cup‐shaped hydrogels.^[^ ^28^ ^]^ Considering that spheroid growth can yield heterogeneous regions of cellular density due to spatial variations in oxygen and nutrient availability,^[^ ^32^ ^]^ we further assessed the local cellular density and morphology in the core and peripheral regions of the spheroids. As no significant differences in either of these variables were observed, we concluded that the distinct growth dynamics of the unconfined and confined spheroids did not affect differential cell aggregation. Qualitative assessment of the spheroid morphology also showed similarity between the unconfined and confined spheroids (Videos S1 and S2, Supporting Information). Crucially, these data established the feasibility of utilizing a direct comparison between unconfined and confined spheroids, taken after five days of culture, to isolate the effect of confinement at the breast cancer interface.

### 2.2 Physical Confinement Within Confined Spheroids Resulted in Lower Sensitivity to Cytotoxic Drugs

**Figure 3:**
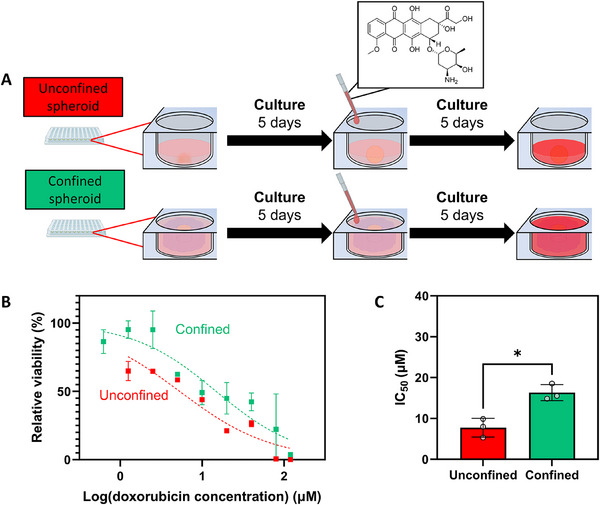
Sensitivity of unconfined and confined spheroids to cytotoxic drug doxorubicin. A) Schematic of doxorubicin drug interrogation. MCF‐7 cells were allowed to aggregate in unconfined and confined conditions for five days prior to exposure of doxorubicin for five further days, including a media change with fresh doxorubicin after two days of exposure. B) Dose‐response curves demonstrating doxorubicin‐dose dependent MCF‐7 cell survival after five days of drug interrogation in unconfined and confined spheroids. Spheroids were cultured in media without any doxorubicin as a 100% viability control to which the measured viability in the drug‐exposed samples could be normalized. Spheroids were cultured in media with 30%w/v ethanol as a positive control for cell death to establish a background signal at 0% viability. Trendlines were derived from a logarithmic variable slope non‐linear regression curve fitting. C) Half‐maximal inhibitory concentration (IC_50_) was extrapolated from trendlines across three experimental repeats and averaged to compare drug sensitivity in unconfined and confined spheroids. Independent sample t‐test between experimental groups. Statistical differences are depicted with ^*^ (0.05 <*p*). Graphs show mean ± SD. N = 3.

To study the effect of physical confinement on drug resistance, MCF‐7 spheroid's sensitivity to chemotherapeutics was probed by culturing MCF‐7 cells under unconfined and confined conditions for five days, with subsequent drug exposure for five further days (**Figure** **3**A). Doxorubicin, an inhibitor of the topoisomerase II cleavage complex responsible for relaxing DNA structures during cell proliferation, was chosen as it is a chemotherapeutic agent commonly used in breast cancer for inducing apoptosis through promoting DNA single‐ and double‐strand breaks.^[^ ^33^, ^34^ ^]^ Across the dose‐response curves, confined spheroids demonstrated higher cell survival as compared to unconfined spheroids. Physical confinement was thus found to reduce the sensitivity of MCF‐7 spheroids to doxorubicin (Figure 3B). Notably, the effect of confinement on cell survival was the most pronounced in the lower end of the dose‐response curve (≤2.5 µm doxorubicin), potentially pointing toward drug resistance mechanisms allowing cells to evade cell death at this range of concentrations. The trends in the dose‐response curves for unconfined and confined spheroids were reflected in the half‐maximal inhibitory concentrations extracted from curve fittings to the dose‐response curves (IC_50_, Figure 3C). Confined spheroids demonstrated a nearly two‐fold increase in IC_50_ as compared to unconfined spheroids (7.74 ± 2.29 µm and 16.31 ± 1.97 µm, respectively, *p* = 0.104), pointing toward physical confinement as a significant contributor in determining the overall cell survival in response to doxorubicin. Additionally, the confinement‐related change in drug sensitivity was not unique to doxorubicin as similar trends were observed when exposing unconfined and confined MCF‐7 spheroids to estrogen receptor‐targeting drug tamoxifen (a two‐fold increase in IC_50_, Figure S7, Supporting Information due to spheroid confinement).

### 2.3 The Hydrogel did not Act as a Drug Diffusion Barrier to Confined Spheroids

**Figure 4:**
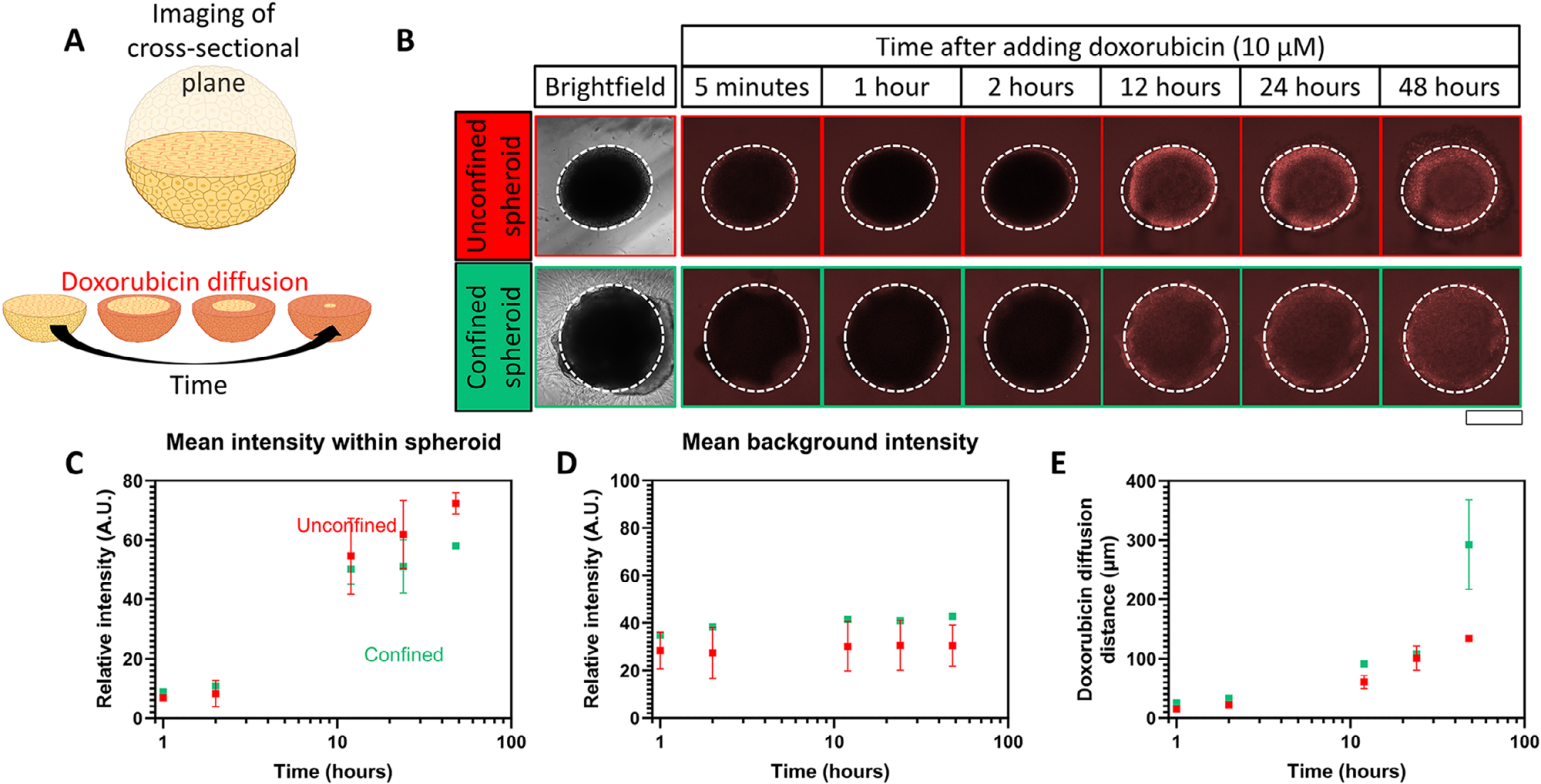
Doxorubicin diffusion kinetics in unconfined and confined spheroids. A) Schematic of cross‐sectional imaging plane for doxorubicin drug diffusion. B) Doxorubicin (10 µm) diffusion through unconfined and confined spheroids tracked through intermittent fluorescent imaging of the medial cross‐sectional area of the samples. The inherent fluorescent properties of doxorubicin (λ_ex_/ λ_em_ = 480/590 nm) to track doxorubicin penetration to the spheroid, and diffusion of the drug into the spheroid. A spheroid perimeter was identified through brightfield imaging and outlined with gridlines. C–E) quantified outputs of diffusion kinetics extracted from fluorescence tracking of doxorubicin during drug exposure. (C) Overall fluorescence intensity of doxorubicin within the spheroid perimeter, as determined by the location of the gridlines, over the drug interrogation period of 48 h. (D) Overall fluorescence intensity of doxorubicin outside the spheroid perimeter, as determined by the location of the gridlines, over the drug interrogation period of 48 h. This region was quantified in unconfined spheroids to track doxorubicin availability within the media. Quantification of this region in confined spheroids was performed to assess the penetration of doxorubicin through the hydrogel. (E) Doxorubicin diffusion distance through spheroids as a function of drug interrogation time. The drug diffusion distance was defined as the longest distance that the drug had diffused into the spheroid from the spheroid perimeter at a given time. Scale bars = 1 mm. Graphs show mean ± SD. N = 3. The figure contains elements created with BioRender.com.

The ability of the hydrogel to act as a drug diffusion barrier to restrict drug access to the tumor cells was probed as the potential underlying cause for the observed differences in drug sensitivity between unconfined and confined spheroids. The inherent fluorescent properties of doxorubicin were exploited to probe doxorubicin diffusion through imaging the median cross‐sectional area of spheroids using confocal microscopy (**Figure** **4**A). Doxorubicin diffusion was tracked intermittently during drug interrogation (10 µm doxorubicin, 48 h, Figure 4B). The fluorescence intensity at the interface of the spheroid and its direct surroundings were of similar strengths in unconfined and confined conditions after just 5 min of incubation (Figure 4C). Moreover, the fluorescence intensity in the background region was constant over time (Figure 4D), suggesting immediate access of doxorubicin to the spheroid interface in both unconfined and confined conditions. This outcome aligned with previous work that observed doxorubicin diffusion up to at least 300 µm in alginate hydrogels within a day of incubation,^[^ ^35^ ^]^ and up to 1000 µm in agarose hydrogels within 10 min.^[^ ^36^ ^]^ Doxorubicin accumulation around the spheroid edges was observed in both unconfined and confined conditions after 12 h of incubation, with doxorubicin progressively diffusing further into the spheroids with time over the monitored drug penetration study. Accumulation of doxorubicin within the unconfined and confined spheroids interestingly did not differ (Figure 4C). Doxorubicin diffusion distance meanwhile was not affected by the extent of confinement up to 24 h of incubation (Figure 4E). Whilst there was a slight difference after 48 h of incubation, this difference may arise from the complexity of the dynamic cellular response to doxorubicin cytotoxicity, including drug accumulation in responsive cells and drug efflux from resistant cells, rather than differences in the drug diffusivity. This poorly understood molecular and cellular interplay is complex and requires further work to elucidate. Overall, these data demonstrated that the differences in drug sensitivity established between unconfined and confined spheroids could not be attributed to any restrictions in drug access or drug diffusion.

### 2.4 Emergence of Phenotypic Changes in Cells within Peripheral Regions of Confined Spheroids

**Figure 5:**
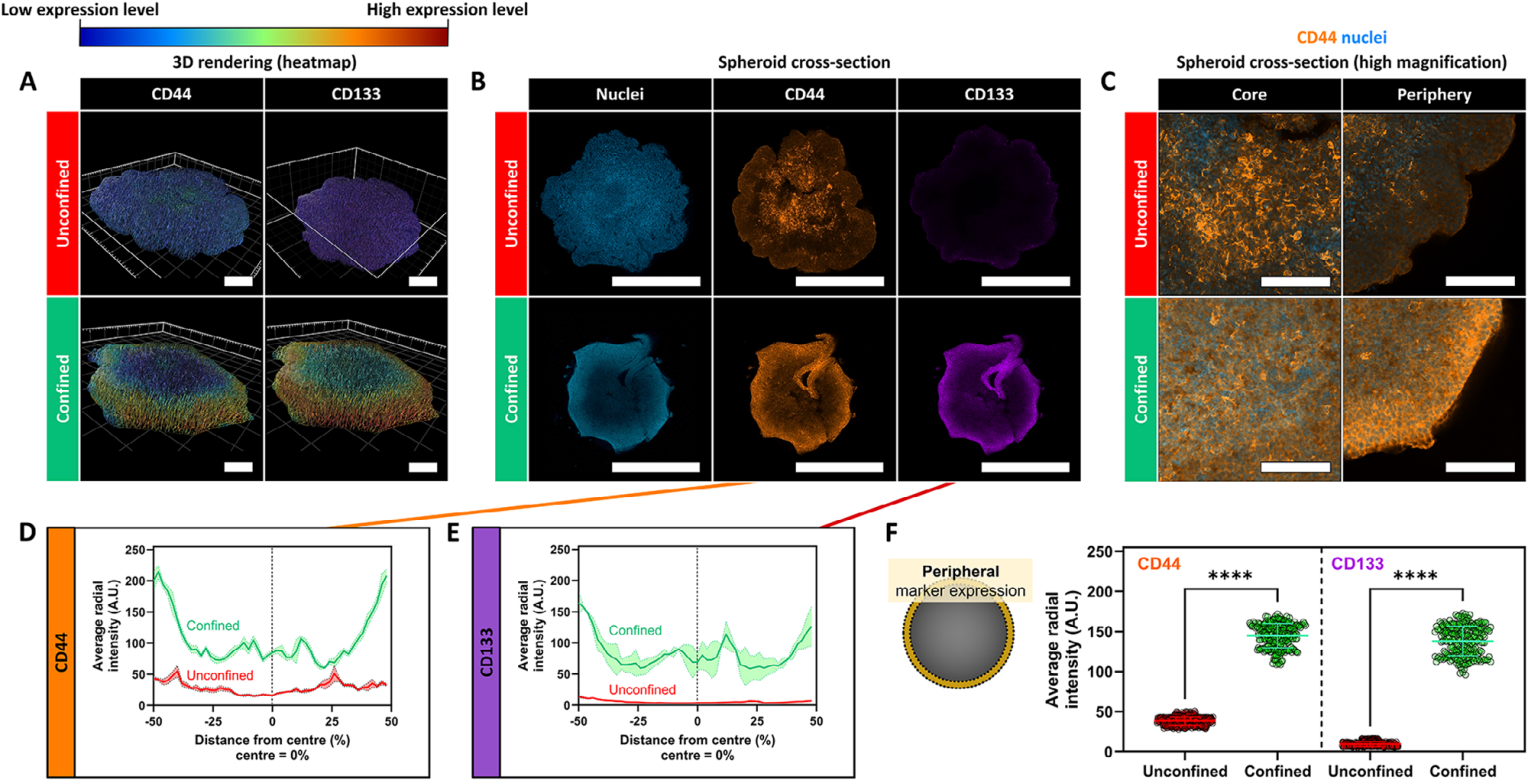
The emergence of cellular heterogeneity at the hydrogel‐spheroid interface. A–C) Representative images of immunofluorescence staining of CD44 (orange) and CD133 (red) in unconfined and confined spheroids. Representative images were displayed as 3D renderings showing the raw images and heatmaps of each image to visualize spatial differences across the spheroid radius (A), maximum intensity projections of the slices at the median of spheroids (B), as well as higher magnifications of peripheral regions of the maximum intensity projections (C). D–F) Quantification of spatial heterogeneity in CD44 (D) and CD133 (E) expression as quantified through the relative fluorescence intensity from immunofluorescence‐stained images. The fluorescence intensity was quantified across the cross‐sectional area and expressed as the average radial fluorescence intensity defined as the fluorescence intensity at a given percentual distance from the peripheries of the spheroid toward the spheroid core. (F) Fluorescence intensities were compared between peripheral regions of unconfined and confined spheroids, defined as the outside 5% sections of the total diameter. Each individual point represents the pixel fluorescent intensity at the set distance within the cross‐sectional area, averaged across the analyzed spheroids. Independent sample t‐test between experimental groups. Statistical differences are depicted with ^****^ (0.0001 <*p*). Scale bars = 200 µm (A), 1 mm (B). Graphs show mean ± SD. N = 3.

It is possible that physical confinement in the confined spheroids drove a solid stress‐induced change in drug sensitivity by priming drug‐resistant cell populations. To probe this hypothesis, two markers (cluster of differentiation 44; CD44,^[^ ^37^ ^]^ cluster of differentiation 133; CD133^[^ ^38^, ^39^ ^]^) were investigated that are positively associated with drug resistance in breast cancer cells. Unconfined and confined spheroids demonstrated spatial heterogeneity in CD44 and CD133 expression throughout 3D heatmap reconstructions of these sample groups (**Figure** **5**A). 3D projections (Videos S1–S4, Supporting Information) verified that the heterogeneity in CD44 and CD133 expression arose mainly in the *X*‐*Y* axis rather than in the Z dimension. This heterogeneity was more clearly visualized through medial sections of these spheroids (Figure 5B,C); in unconfined spheroids, breast cancer cells demonstrated low, diffuse CD44 expression and predominantly lacked CD133 expression. Quantitative analysis of these expression patterns revealed that biomarker expression for unconfined spheroids was similar across the spheroid radius (Figure 5D,E). In contrast, significant upregulation of membrane‐bound CD44 and CD133 was observed at the periphery of confined spheroids (Figure 5F). Co‐occurrence of CD44 and CD133 was furthermore apparent in these peripheral populations (Figure 5B), pointing toward the emergence of a CD44^+^ CD133^+^ breast cancer population at the periphery of the confined spheroids. Early signs of the emergence of the CD44^+^ CD133^+^ population were apparent already after one day of culture in the confined condition, supporting the notion that the MCF7 cells experienced confinement of the surrounding alginate hydrogel at these early stages of growth (Figure S8, Supporting Information). CD44 and CD133 gradually increased over the culture period, yielding the CD44^+^ CD133^+^ cells at the periphery of confined spheroids after seven days of culture that demonstrated a 3.8‐ and 14.3‐fold increase in fluorescence intensity as compared to the equivalent regions in unconfined spheroids for CD44 and CD133, respectively). These peripheral regions were notably exposed to a different environment in unconfined and confined conditions. Cells at the spheroid periphery were in contact with either media or the alginate hydrogel in unconfined and confined conditions, respectively. It should be noted that spheroids were only confined laterally, with free access to media in the axial direction. Lateral confinement by the hydrogel cup was sufficient to affect a reduction in drug sensitivity as the addition of a confining lid on top of the cavity did not yield a further reduction in drug sensitivity. The concurrent peripheral upregulation of CD44 and CD133 was also observed in the fully confined spheroids (Figure S9, Supporting Information). Differences in oxygen and nutrient diffusion between unconfined and confined conditions may also contribute to driving cellular heterogeneity.^[^ ^32^ ^]^ While we did not observe significant differences in the hypoxia‐inducable factor 1‐alpha (HIF‐1α, Figure S10, Supporting Information), there is likely an interplay between hypoxia and confinement at play within native cancers. Within this work, we focused on understanding confinement as a key factor in inducing the upregulation in biomarker expression in the peripheral regions of the confined spheroids.

### 2.5 Confinement‐Induced Peripheral Cellular Plasticity Correlated with an Increase in Drug Resistance

**Figure 6:**
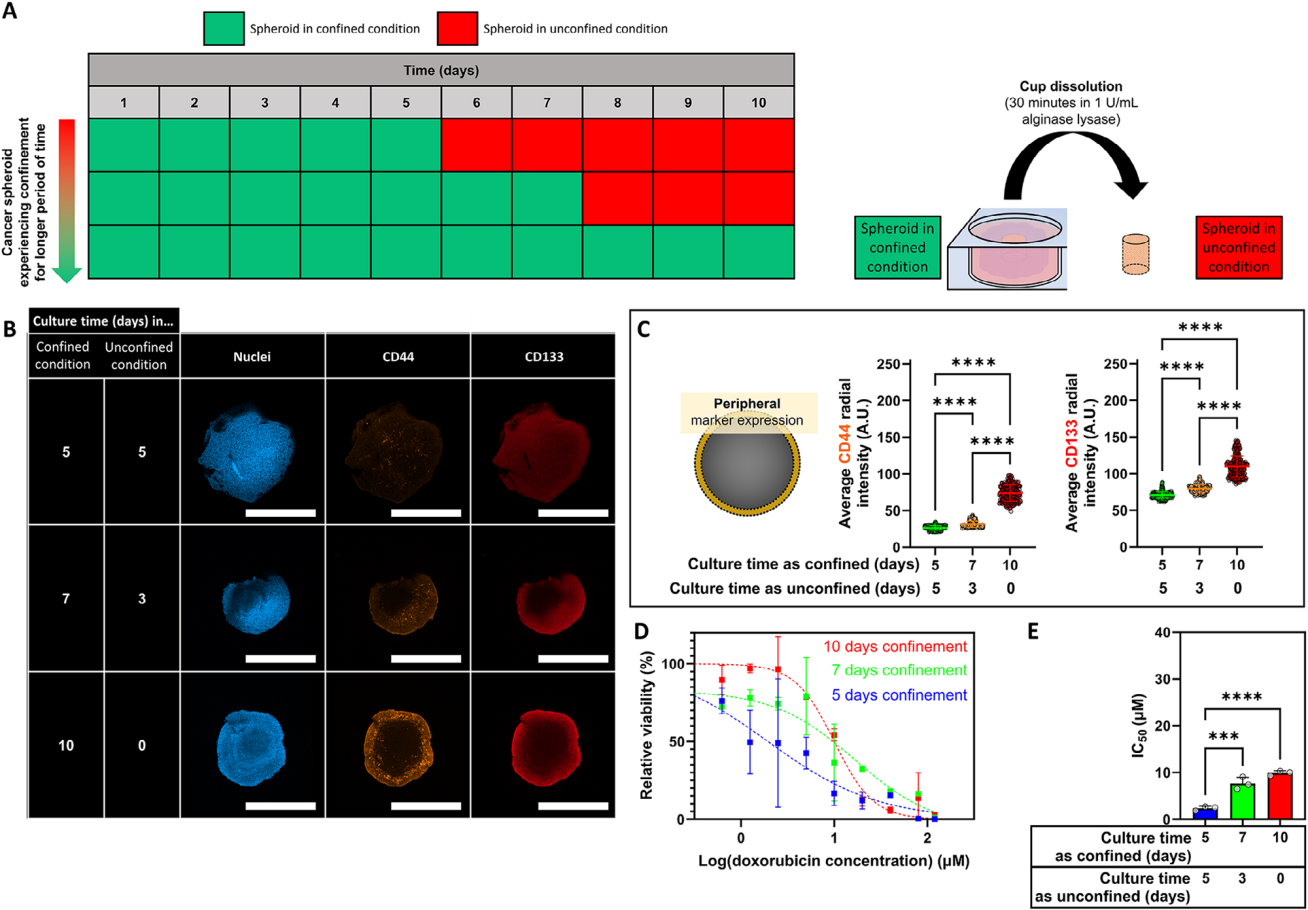
Modulating the period of spheroid confinement to evaluate the link between confinement and drug resistance. A) Schematic of the timeframe of confinement. Spheroids were cultured for a total of 10 days, but the period in which spheroids experienced confinement varied between the groups. To alter the period of confinement, the hydrogel cup was enzymatically degraded at the appropriate time points. B) Representative images of immunofluorescence staining of CD44 (orange) and CD133 (red). Representative images were displayed as maximum‐intensity projections of the slices at the median of spheroids. C) Quantification of spatial heterogeneity in CD44 and CD133 expression. Fluorescence intensities were determined in the spheroid's peripheral regions, defined as the outside 5% sections of the total diameter, respectively. Each individual point represents the pixel fluorescent intensity at the set distance within the cross‐sectional area, averaged across the analyzed spheroids. D,E) Dose‐response curves (D) and half‐maximal inhibitory concentration (IC_50_) curves (E) demonstrating doxorubicin‐dose dependent MCF‐7 cell survival after five days of drug interrogation in unconfined and confined spheroids. Spheroids were cultured in media without any doxorubicin as a 100% viability control to which the measured viability in the drug‐exposed samples could be normalized. Spheroids were cultured in media with 30%w/v ethanol as a positive control for cell death to establish a background signal at 0% viability. One‐way ANOVA with Tukey post‐hoc test to compare multiple experimental groups. Independent sample t‐test between experimental groups. Statistical differences are depicted with ^***^ (0.001 <*p*) and ^****^ (0.0001 <*p*). Scale bars = 1 mm. Graphs show mean ± SD. N = 3.

The potential relation between drug resistance and the emergence of cellular plasticity in peripheral regions observed in confined spheroids was investigated next. We hypothesized that the emergence of drug resistance in the confined spheroids was the result of continuous physical confinement from the hydrogel cup. To test this hypothesis, the time during which the spheroid experienced confinement was varied between experimental groups. The downstream effect on drug resistance markers and drug sensitivity was then determined as a function of the duration of spheroid confinement. The duration of confinement could be controlled by dissolving the surrounding hydrogel cup at different time points during the culture period (**Figure** **6**A), allowing the spheroids to grow in unconfined conditions for the remainder of the culture period. Interestingly, the period of spheroid confinement was observed to positively correlate to the CD44 and CD133 marker expression at the spheroid peripheries (Figure 6B); whereas a CD44^+^ CD133^+^ cell sub‐population was observed in spheroids that experienced 10 days of confinement (CD44: 73.6 ± 11.1 mean intensity (A.U.), CD133: 109.9 ± 14.4 mean intensity (A.U.), Figure 6C), reducing the period of confinement to the initial five days of culture led to a significant decrease in drug‐resistant marker expression (CD44: 26.3 ± 2.8 mean intensity (A.U.), CD133: 70.8 ± 4.4 mean intensity (A.U.), *p* <0.0001, Figure 6C). In addition to these observations, we noted that the CD44 and CD133 fluorescence intensities in peripheral regions of spheroids stained after 5 days in confinement (Figure S8, Supporting Information) were ≈6‐ and ≈2‐fold higher, respectively than in spheroids that experienced 5 additional culture days without confinement prior to staining (Figure 6C). Collectively, these data established spheroid confinement as the cause for the emergence of the peripheral CD44^+^ CD133^+^ populations. To further probe whether the confinement‐induced emergence of these putative drug‐resistant cell populations affected change in overall spheroid drug sensitivity, spheroids were exposed to doxorubicin (0–120 µm). Spheroids were exposed to doxorubicin between days 6 and 10 post‐fabrication, whilst the period of spheroid confinement was varied between experimental groups to five, seven, and ten days. Spheroids that experienced longer periods of confinement demonstrated decreased drug sensitivity (Figure 6D), affecting a trend toward up to a 5‐fold increase in IC_50_ concentration in spheroids grown in confinement for longer periods of time (5 days: 1.9 ± 0.9 µm, 10 days: 10.0 ± 1.7 µm, *p* <0.01, Figure 6E). Taken together, a concurrent dependency of the period of confinement on the expression levels of peripheral biomarkers related to drug resistance and the sensitivity of spheroids to cytotoxic drug doxorubicin was uncovered. Inadvertently, these results demonstrated that spheroid confinement from the hydrogel cups was responsible for the emergence of a drug‐resistant CD44^+^ CD133^+^ cancer cell population.

### 2.6 Mechanotransduction Pathways were Implicated in Confinement‐Induced Drug Resistance

**Figure 7:**
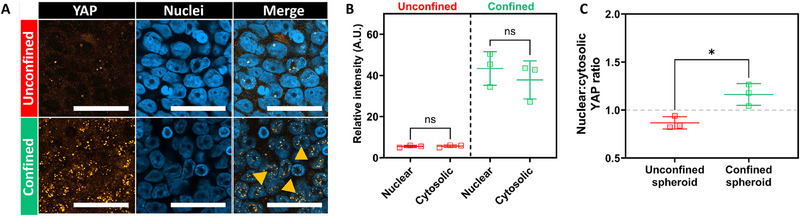
Confinement‐induced nuclear accumulation of mechanotransduction marker YAP. A) Representative images of immunofluorescence staining of YAP (orange) and nuclei (blue). Representative images were displayed as single‐slice images from the periphery of unconfined and confined spheroids. B,C) Quantification of the average YAP fluorescence intensity in nuclear and cytosolic regions (C), as well as the nuclear‐to‐cytosolic ratio of YAP fluorescence intensity as determined for each individual image. One‐way ANOVA with Tukey post‐hoc test to compare multiple experimental groups. Independent sample t‐test between experimental groups. Statistical differences are depicted with ^*^ (0.05 <*p*), and statistical non‐significance was indicated with (ns = not significant). Scale bars = 50 µm. Graphs show mean ± SD. N = 3.

The cellular mechanism behind the confinement‐induced drug resistance observed in breast cancer cells present at the interface between the cancer spheroids and the hydrogel cups was explored. We hypothesized that the physical confinement by the hydrogel cups led to accumulation of solid stress at this interface. The yes‐associated protein (YAP) is a central driver in solid stress initiation of cellular signaling cascades. YAP expression was therefore probed at the interfaces of confined and unconfined spheroids (**Figure** **7**A,B).

**Figure 8:**
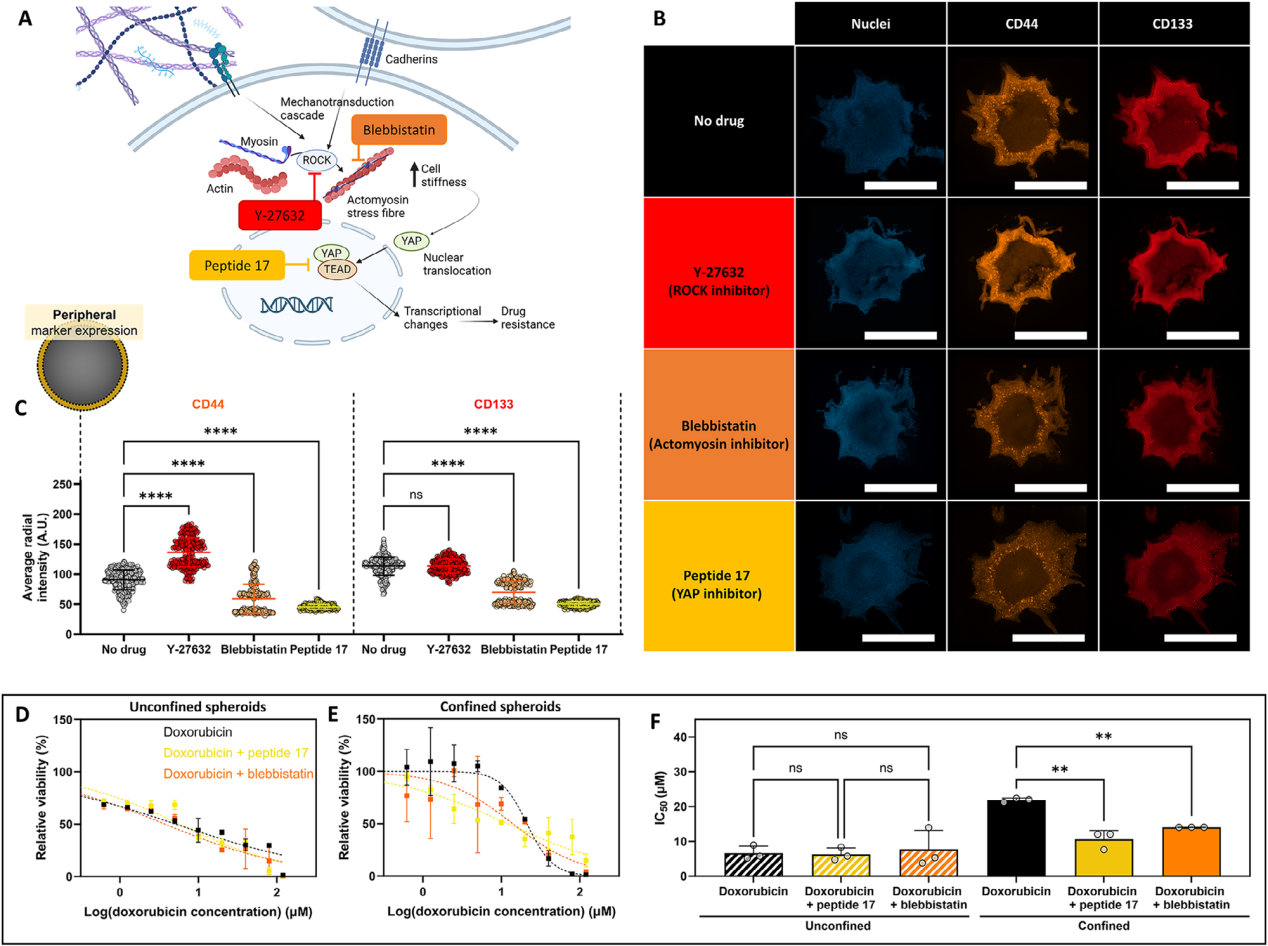
The mechanotransduction pathway was implicated in confinement‐induced drug resistance. A) Schematic showing key parts of the mechanotransduction pathway in driving transcriptional changes in cancer cells. B) Representative images of immunofluorescence staining of CD44 (orange) and CD133 (red). Representative images were displayed as maximum‐intensity projections of the slices at the median of spheroids. C) Quantification of spatial heterogeneity in CD44 (C) and CD133 D) expression. Fluorescence intensities were determined in the spheroid's peripheral regions, defined as the outside 5% sections of the total diameter, respectively. Each individual point represents the pixel fluorescent intensity at the set distance within the cross‐sectional area, averaged across the analyzed spheroids. (D,E) Dose‐response curves for unconfined (D) and confined E) spheroids. F) Half‐maximal inhibitory concentration (IC_50_) curves (F) demonstrating doxorubicin‐dose dependent MCF‐7 cell survival after five days of drug interrogation in unconfined and confined spheroids. Spheroids were cultured in media without any doxorubicin as a 100% viability control to which the measured viability in the drug‐exposed samples could be normalized. Spheroids were cultured in media with 30%w/v ethanol as a positive control for cell death to establish a background signal at 0% viability. One‐way ANOVA with Tukey post‐hoc test to compare multiple experimental groups. Independent sample t‐test between experimental groups. Statistical differences are depicted with ^**^ (0.01 <*p*), and statistical non‐significance was indicated with (ns = not significant). Scale bars = 1 mm. Graphs show mean ± SD. N = 3.

We observed intranuclear accumulation of YAP exclusively in the confined conditions, which was indicative of YAP activation in this condition (Figure 7C). While it should be noted that nuclear accumulation is not exclusive to solid stress‐driven processes, we conclude that cells likely experienced solid stress under confinement that contributed to phenotypic changes within the peripheral cell populations. Other processes that affect YAP translocation, such as hypoxia‐induced signaling^[^ ^40^ ^]^ and cell cycle positions,^[^ ^41^ ^]^ may contribute to the confinement‐induced cellular response. Cells may respond to solid stress through mechanotransduction pathways that lead to changes in cellular protein expression that may underline the observed emergence of drug resistance. To test this hypothesis, three key proteins involved in mechanotransduction (ROCK, myosin II, and YAP) were targeted through pharmacological intervention during spheroid growth (**Figure** **8**A).

The confinement‐induced emergence of CD44^+^ CD133^+^ breast cancer cell populations was still observed in peripheral regions of cancer spheroids after pharmacological inhibition of ROCK, myosin II, and YAP. However, the CD44 and CD133 expression levels were found to be reduced (Figure 8B,C) after pharmacological inhibition of myosin II (with blebbistatin treatment) and YAP (with peptide 17 treatment) but not upon ROCK inhibition (with Y‐27632 treatment). Notably, the reduction in CD44 and CD133 expression was independent of spheroid growth kinetics (Figure S11, Supporting Information). Blebbistatin inhibits the complementation of cytosolic actin and myosin to form actomyosin stress fibers, whilst peptide 17 impedes YAP‐induced transcriptional changes further downstream the mechanotransduction pathway. These stress fibers increase cellular tension and allow force transmission of external forces toward the nucleus to drive transcriptional changes. Y‐27632 can impede this pathway through the inhibition of ROCK‐mediated actomyosin stress fiber formation. It could therefore be concluded that confinement‐induced emergence of the CD44^+^ CD133^+^ breast cancer cell population was at least in part mediated through ROCK‐independent mechanotransduction.

We successfully linked the confinement‐induced emergence of a CD44^+^ CD133^+^ breast cancer cell population at the spheroid periphery to increased drug resistance. To confirm the underlying involvement of mechano‐sensation within drug resistance, the sensitivity to chemotherapeutic agent doxorubicin was probed in spheroids treated with peptide 17 and blebbistatin (Figure 8D–F). The drug sensitivity of unconfined spheroids was not affected by the pharmacological intervention of peptide 17 (IC_50_: 7.05 ± 1.81 µm, *p* >0.9999) and blebbistatin (IC_50_: 8.88 ± 7.20 µm, *p* = 0.9908), indicating YAP‐ and myosin‐mediated cell signaling pathways were not involved in driving drug resistance in the absence of confinement. In contrast, pharmacological intervention with peptide 17 and blebbistatin within confined spheroids impeded confinement‐induced drug resistance as evidenced by a nearly two‐fold decrease in doxorubicin IC_50_ within confined spheroids treated with peptide 17 (IC_50_: 9.86 ± 3.13 µm, *p* = 0.0121) and blebbistatin (IC_50_: 14.06 ± 0.02 µm, *p* = 0.0240) as compared to untreated spheroids (IC_50_: 21.86 ± 0.58 µm). Taken together, these observations verified a key link between physical confinement and the emergence of a drug‐resistant CD44^+^ CD133^+^ breast cancer cell population at the cancer spheroid peripheries.

## 3 Discussion

Our study demonstrates how the physical confinement of tumor tissue by a surrounding matrix‐mimicking hydrogel induces multidrug resistance in breast cancer, likely through solid stress‐induced cell signaling. Other studies have probed the effects of solid stress on cancer mechanisms through adjusting hydrogel stiffness in spheroid‐embedded hydrogels.^[^ ^23^, ^26^, ^42^ ^]^ Increased solid stress in stiffer hydrogels was found to affect spheroid size,^[^ ^23^ ^]^ stemness markers,^[^ ^26^, ^42^ ^]^ and malignancy.^[^ ^42^ ^]^ Within these studies, solid stress‐driven elevation in biomarker expression crucially occurred homogeneously throughout the spheroids. In contrast, our work here reports spheroids that distinctively demonstrate cellular heterogeneity as evidenced by the emergence of a CD44^+^ CD133^+^ drug‐resistant niche at the periphery of the cancer spheroids. Cellular heterogeneity has been observed in peripheral regions of cancers within clinical and animal in vivo studies across a range of cancer types including glioma,^[^ ^43^ ^]^ colon carcinoma^[^ ^44^ ^],^ and hepatocellular carcinoma.^[^ ^45^ ^]^ Notably, stemness markers such as CD133 have also been found to be upregulated in the peripheral regions of cancers in in vivo xenografts.^[^ ^44^ ^]^ Whilst further work is required to evidence the presence of the peripheral drug‐resistant niche in breast cancer, the cellular heterogeneity observed within our model appears to align in part with the cellular composition found in native cancers. The disparity in structural composition between embedded spheroid models and our work may account for the lack of heterogeneity observed within typical 3D in vitro models. Space restriction in embedded spheroids leads to a decrease in proliferation and the homogeneous accumulation of compressive stress within the tumors.^[^ ^46^ ^]^ An equilibrium between the expanding spheroid and the restricting matrix furthermore links spheroid size to the stiffness of the embedding matrix; increased matrix stiffness leads to a reduction in proliferation^[^ ^24^ ^]^ and overall spheroid size.^[^ ^24^, ^47^ ^]^ This contrasts the native tumor growth stages during which tumor size increases as the surrounding matrix densifies. Additionally, solid stress‐dependent heterogeneity assessment using this approach can be confounded by paracrine signals between multiple matrix‐bound aggregates. The drop‐on‐demand printed spheroid model used in our work is a unique approach wherein aggregate formation and stresses from confinement can be independently controlled to test structure‐function relationships at the interfacial mimic of ductal adenocarcinoma. Here, we propose that the mimicry of the cancer‐host tissue interface anatomy was crucial in replicating solid stress regimes. The mimicry of this stress regime was in turn imperative in driving cellular heterogeneity and the emergence of the peripheral drug‐resistant cellular niche. We hypothesize that drop‐on‐demand printing will enable tailoring of the physicochemical properties of the confining hydrogel cups. Our study evaluated the extremes of “confined” versus “unconfined,” thereby laying the groundwork in which subtle differences in confinement may be probed, opening up new avenues to systematically tailor the biophysical and biochemical properties of an encapsulating matrix. In contrast to other natural hydrogel platforms, these properties can easily be tailored in alginate‐based systems.^[^ ^48^, ^49^, ^50^ ^]^ Our model may thus be ideally suited to understand the role of matrix composition and factors such as viscoelasticity in driving solid stress‐mediated cellular plasticity at the cancer‐host tissue interface.

Drug resistance is classically viewed as hypoxia‐driven metabolic and transcriptional changes that occur within the core of spheroids and tumoroids; the so‐called “hypoxic niche.”^[^ ^51^ ^]^ This establishment of a hypoxic niche is in part driven by solid stress restricting blood vessels in the breast tissue, whilst the buildup of intracellular stress within the tumor core will drive hypoxia‐inducible factor 1‐alpha (HIF1α) signaling, thereby linking solid stress to hypoxia.^[^ ^52^ ^]^ Several recent studies have identified the potential of an “invasive niche” within a range of cancers such as glioblastoma,^[^ ^53^, ^54^ ^]^ melanoma^[^ ^55^ ^]^ and in breast tissue.^[^ ^56^ ^]^ Cancer cells with high plasticity have been identified within this niche that are thought to play a key role in malignancy. Here, we present evidence that physical confinement can catalyze the emergence of a drug‐resistant population at the invasive niche located at the periphery of a growing tumor. This relationship has been probed previously within our group using 2D cancer models.^[^ ^19^, ^20^, ^55^ ^]^ Our work builds on this body of literature by demonstrating the existence of the peripheral drug‐resistant cellular niche within a 3D context. This drug‐resistant niche may carry additional significance due to similarities with the invasive niche concept, linking solid stress to pro‐metastatic behavior. Further study of the hypoxic and peripheral drug‐resistance cell niches, their respective functions, interplay, and differences, will be important for future therapeutic strategies to impede drug‐resistant breast cancers.

It is well‐established that the emergence of drug resistance can be coordinated through cell adhesion to matrix proteins within the extracellular matrix.^[^ ^57^, ^58^, ^59^, ^60^ ^]^ Here, we used alginate, which is devoid of adhesive sequences. We thus observed a distinct adhesion‐independent confinement‐mediated pathway for the emergence of drug resistance. Joyce et al. investigated the dependency of doxorubicin resistance on the surrounding matrix in breast cancer cell‐embedded alginate hydrogels.^[^ ^61^ ^]^ Dynamically increasing hydrogel stiffness, which increased stress exerted onto the cancer cells, increased the overall drug resistance of encapsulated MDA‐MB‐231 cells. In contrast to our study, adjusting the extent of stress did not yield an increase in drug resistance in MCF‐7 cells.^[^ ^61^ ^]^ While the reason for this is unclear, there are key differences in this work^[^ ^61^ ^]^ compared to ours. Spheroid size varied significantly depending on the hydrogel stiffness, with stiffer matrices generally leading to smaller spheroids due to an equilibrium shift between proliferation‐mediated pressure and counteracting compressive forces from the surrounding matrix. This shift emulates native cancer progression, wherein cancer size increases in tandem with an increase of matrix stiffness through fibrosis/desmoplasia. We control the MCF‐7 spheroid size in our model, thus providing the means to investigate the effect of physical confinement in a size‐independent manner. This control allowed us to reveal a relationship between solid stress and the emergence of an interfacial drug‐resistant population. Future work may aim to elucidate the contribution of bioactivity in this relationship by exploiting peptide‐conjugated bioinks that have been developed for drop‐on‐demand printing within our research group.^[^ ^31^ ^]^

Rather than direct adhesion to the surrounding material, solid stresses exerted by the matrix was presumed to have caused the accumulation of intertumoral stress propagated through cell‐cell junctions. Pharmaceutical inhibition of YAP and actomyosin contraction were effective in impeding mechanotransduction and the subsequent emergence of drug resistance. While ROCK inhibition in our study did not lead to a significant change in drug‐resistant phenotypes, several reports have indicated ROCK involvement in drug resistance. Common to these studies is the important role of ROCK during proliferation, where inhibition presumably impedes the enrichment of drug‐resistant progeny,^[^ ^14^, ^23^ ^]^ potentially in a stress‐dependent manner.^[^ ^62^ ^]^ In contrast, we control proliferation in our drop‐on‐demand printed spheroids so that the direct effects of physical confinement on a tumor model can be probed. Therefore, it is tempting to speculate that the drug‐resistant phenotype observed here is related to the proliferation‐independent reprogramming of a subpopulation to a drug‐resistant state. Nevertheless, more work is required to decouple the role of proliferation and the influence of solid stress on adaptation to drug‐resistant states.

A striking observation in our study was the full reversal of the confinement‐mediated increase in drug resistance in our model upon YAP inhibition. There is a body of work linking YAP expression to drug resistance across a range of cancer types such as breast^[^ ^63^ ^]^ and lung cancer.^[^ ^64^ ^]^ Clinically, breast cancers with higher overall YAP expression demonstrate a trend toward reduced survival due to increased drug resistance.^[^ ^65^ ^]^ Consequently, YAP inhibitors have been tested as candidate drugs for drug‐resistant breast cancer. Verteporfin targets the YAP complexation with the transcription‐enhanced association demain (TEAD), reducing drug resistance in vitro. In vivo effect however requires high verteporfin concentrations with off‐site cytotoxicity,^[^ ^66^, ^67^ ^]^ preventing its use in a clinical setting. It is suggested that the inability to target drug‐resistant populations with upregulated YAP within the total cancer population may underline the limited success of verteporfin in clinical testing.^[^ ^68^ ^]^ Improving our understanding of the downstream interactions of YAP within drug‐resistant cancer niches is thus crucial in identifying ways to target this key protein, or upstream targets, in breast cancer. With these goals in mind, our confined spheroid model replicates a YAP inhibitor‐responsive drug‐resistant cancer population and may therefore be ideally placed to further probe the roles of YAP in breast cancer drug resistance. In line with this direction, potential dynamic changes in cellular phenotype in response to chemotherapeutic drug exposure,^[^ ^69^, ^70^ ^]^ either with or without preceding YAP inhibition, should be determined in follow‐up work.

## 4 Conclusion

Our study sheds light on the emergence of a drug‐resistant peripheral cellular niche at the interface of cancer and its neighboring environment. This finding complements the existing paradigm around drug resistance largely stemming from hypoxia‐related signaling in oxygen‐depleted regions of breast cancers. The drop‐on‐demand printed model used within this study provides a unique reductionist tool to probe the mechanisms behind confinement‐induced drug resistance within this peripheral niche. This approach will support efforts to unravel the key contributors to cancer progression, such as the mechanobiological basis to drug resistance highlighted in this study. Moving forward, we expect that this model will allow further systematic investigations into the interactions that occur at the interface between cancers and their microenvironment.

## 5 Experimental Section

### Materials

High‐glucose Dulbecco's modified eagle medium (DMEM), Dulbecco's phosphate buffered saline (PBS), Hank's buffer solution, trypsin‐EDTA 0.25%w/v, penicillin‐streptomycin (10 000 units mL^−1^; PS), Triton X‐100, phalloidin Atto 488, 4′,6‐diamidino‐2‐phenylindole (DAPI), primary antibody against CD44 (MA513890), and donkey antibody AlexaFluor 555‐conjugated anti‐mouse secondary antibody were purchased from ThermoScientific (Australia). Calcein‐AM, propidium iodide, porcine skin type A gelatin (bloom strength 300 g), bovine serum albumin (BSA), alginate lyase, doxorubicin hydrochloride, triethylamine, goat antibody AlexaFluor 647‐conjugated anti‐rabbit secondary antibody, and Y‐27632 were purchased from Sigma‐Aldrich (Australia). Calcium chloride, urea, and sucrose were purchased from Chem Supply (Australia). CellTiter‐Glo 3D Viability Assay was purchased from Promega (Australia). Primary antibody against CD133 (MSB462020) was purchased from MyBioSource (CA, USA). A primary antibody against YAP (SC‐101199) was purchased from Santa Cruz. Primary antibody against HIF‐1α (ab51608) and blebbistatin were purchased from Abcam (UK). Peptide 17 was purchased from Selleckchem (TX, USA).

### Cell Culture

MCF7 cells were obtained from the American Type Culture Collection (VA, USA). MCF‐7 cells were cultured in DMEM expansion media supplemented with 10% FBS and 1% penicillin‐streptomycin. Cells were maintained in culture under physiological conditions (37 °C, 5% CO_2_) until confluency, upon which the cells were collected using 0.25% w/v trypsin‐EDTA and sub‐cultured (ratios 1:5–1:15). MCF7 cells were used for experiments between passages 15 and 25.

### Drop‐On‐Demand Printing of Cup‐Shaped Hydrogels

Bioinert cup‐shaped alginate‐based hydrogels were fabricated using a non‐contact drop‐on‐demand printer Rastrum (Inventia Life Sciences, Australia). The fabrication approach was adapted from published work from our group.^[^ ^28^ ^]^ The printer utilized a flyby printhead that sequentially dispensed droplets of calcium chloride (2%w/v in MilliQ water) and alginate (2%w/v in MilliQ water) into each of 12 wells within a row of a 96‐well plate, with reproducible control over droplet size and location due to the printer's pressure regulator and 2‐axis motion control system, respectively. High‐throughput fabrication of cup‐shaped hydrogels was facilitated through an automated layer‐by‐layer approach that involved the printing of a flat alginate‐based bioinert base and the subsequent printing of alginate‐based walls on top of the base to complete a cup‐shaped structure. Hydrogel cup size and shape were derived from Brightfield imaging.

### Unconfined and Confined Spheroid Generation

Unconfined spheroids were generated using a well‐established method based on cell‐cell aggregation within a low‐adherent 96‐well U‐bottom plate. Briefly, each well was filled with 30 µL (1 × 10^4^ cells per well), after which cells were left to aggregate for 2 days prior to detaching the formed pellets through refreshing the culture media. The formed MCF‐7 spheroids were cultured for up to 10 days, changing media every 2–3 days. Following a pre‐established protocol,^[^ ^71^, ^72^ ^]^ media was carefully aspirated upon media changes to prevent disturbing the free‐floating spheroids. Determination of unconfined spheroid volume was derived from the largest spheroid diameter as derived from Brightfield images, assuming a spherical shape.^[^ ^73^, ^74^ ^]^ Spheroids were formed in confinement by dispensing 1 × 10^4^ cells per well in nanolitre‐sized droplets of high‐density MCF‐7 cell suspension (250 × 10^6^ cells mL^−1^) within the cavities of cup‐shaped alginate hydrogels. These spheroids, termed confined spheroids, were cultured for up to 10 days, changing media every 2–3 days. Cell number in unconfined and confined spheroids was determined by quantifying ATP content using a CellTiter‐Glo 3D Viability Assay according to a modified protocol based on the manufacturer's instructions. Briefly, culture media was removed altogether, and CellTiter‐Glo 3D Viability Assay solution (1:4 dilution in PBS) was added onto the spheroids. Samples were centrifuged at 300 rpm for 5 min prior to incubation of the samples in the dark at room temperature for 30 min. Luminescence readings were obtained from individual wells using a ClarioStar Plus microplate reader. MCF7 cell suspensions of known cell density were used as calibrates for calculating cell number.

### Cytotoxic Drug Sensitivity Assay

Doxorubicin stocks were dissolved in PBS, filtered through a 0.22 µm sterile filter, and kept frozen until use. Unconfined and confined spheroids were cultured for 5 days prior to the interrogation of the spheroids by the cytotoxic drug doxorubicin (0–120 µm). After 5 days of culture, culture media was removed and replaced with culture media supplemented with doxorubicin. As a positive control for cell death, spheroids were cultured in media supplemented with 30%w/v ethanol. Spheroids were cultured for 5 further days, refreshing media after 2 days with fresh culture media supplemented with doxorubicin (or ethanol). Cell survival was determined using a CellTiter‐Glo 3D Viability Assay according to a modified protocol based on the manufacturer's instructions. Briefly, culture media was removed altogether, and CellTiter‐Glo 3D Viability Assay solution (1:4 dilution in PBS) was added onto the spheroids. Samples were centrifuged at 300 rpm for 5 min prior to incubation of the samples in the dark at room temperature for 90 min. Luminescence readings were obtained from individual wells using a ClarioStar Plus microplate reader. Drug sensitivity curves were constructed from the averaged luminescence readings from triplicates using Equation (1). Trendlines were extrapolated from each experiment repeat using a logarithmic variable slope non‐linear regression curve fitting in GraphPad Prism software, version 10. From these curves, the half maximal inhibitory concentration (IC_50_; the drug concentration required to cause death to half the cell population) was extracted per experimental repeat, after which the average IC_50_ ± standard deviation was determined for the experimental groups.

(1) $$Relative\;viability\;\%\;=\frac{L-L_{EtOH}}{L_{0}-L_{EtOH}}\times 100\%$$

Equation (1) Calculation of relative viability as a function of luminescence readings obtained from the CellTiter‐Glo 3D Viability Assay. L = luminescence reading obtained from averaged triplicates after interrogation of spheroids with 0 to 120 µm doxorubicin. L_0_ = luminescence reading obtained from averaged triplicates after spheroids unexposed to cytotoxic drugs. L_EtOH_ = luminescence reading obtained from averaged triplicates after spheroids exposed to 30%w/v ethanol.

### Drug Penetration Assay

Unconfined and confined spheroids were cultured under physiological conditions for 5 days prior to interrogation of the spheroids by cytotoxic drug doxorubicin (10 µm). Periodically (after 5 min of incubation, and after 1, 2, 12, 24, 48, and 72 h of incubation), fluorescent images were taken of the medial cross‐sections of each sample, exploiting the fluorescent properties of doxorubicin (λ_ex_/ λ_em_ = 480/590 nm) to investigate the penetration of the cytotoxic drug through the spheroids. Brightfield images were taken at the same time to establish the interface between the spheroid and its surrounding environment. The kinetics of drug penetration were extrapolated from these images using ImageJ. The mean fluorescence intensity was determined within the boundaries of the spheroid, as well as the area outside of this region of interest (referred to as “background”). The average fluorescence intensity within the spheroid region was taken as a measure of drug penetration through unconfined and confined spheroids. The doxorubicin diffusion distance was furthermore determined by measuring the distance from the edge of the spheroid perimeter to the diffusion front of doxorubicin within the spheroid. The average intensity of the background regions surrounding the unconfined spheroids was taken as a measure of the doxorubicin content within the media. For confined spheroids, the rate of drug penetration through the cup‐shaped alginate‐based hydrogel cups surrounding the spheroids could be evaluated through evaluating the change in mean intensity within the background region wherein the alginate hydrogel was located. The rate of drug penetration within this region was compared to the background region intensity of unconfined spheroids, wherein it was assumed that the access of the drug to the unconfined spheroids was unrestricted as there was direct contact between the unconfined spheroid and the culture media containing doxorubicin.

### Immunofluorescent Staining, Imaging and Quantification

Samples were fixed after 7–10 days of culture in 4% w/v formaldehyde for 48 h and washed in PBS. Samples were then permeabilized with 2%w/v Triton X‐100 in PBS for a further 24 h, after which samples were washed with PBS and blocked with 3%w/v BSA for 4 h. Thereafter, samples were washed in PBS and incubated with primary antibody against CD44, CD133, HIF‐1α, and YAP, dissolved in 1%w/v BSA in PBS for 4 days. Samples were again washed in PBS prior to incubation with AlexaFluor 555‐ and AlexaFluor 647‐conjugated secondary antibodies, as well as DAPI and Phalloidin Atto 488 stain solutions, for 4 days. Samples were then washed in PBS and kept at 4 °C until imaging. Prior to imaging, samples were transferred into a glass‐bottom 96‐well plate and immobilized by dropping a small amount of heated (45 °C) 7%w/v gelatin on top of the sample. After letting the gelatin cool, the samples were optically cleared using a clearing agent composed of urea, sucrose, and triethylamine (molar ratio 1:2.9:0.68). Z‐stacks of samples were taken on a Zeiss LSM 800 confocal microscope coupled with Zen Blue software. Images were processed through Imaris (Oxford Instruments) for visualization and quantification of cellular density and morphology. In‐built surface rendering was conducted on the nuclear (DAPI) stains of the spheroid Z‐stacks to identify individual cells. F‐actin staining was used to identify the total spheroid volume within the region of interest, allowing quantification of cellular density by division of total cell number by the spheroid volume. Cellular sphericity, defined by the surface‐to‐volume ratio of the individual cell, was extracted from Imaris as a measure of cellular morphology. The relative fluorescence intensity across the cross‐section of the z‐stacks from unconfined and confined spheroids was determined using a custom‐made macro in Image J (see the Supporting Information, Extended Experimental Section). For this protocol, 15 representative slices of the z‐stack were taken and split into individual images. A 2D graph of pixel intensities was extrapolated from three separate lines drawn across the cross‐sections of each biomarkers within each channel. These plots were then averaged to yield a profile of relative fluorescence intensities across the spheroid cross‐sections for each slice, which were then averaged to yield relative cross‐sectional fluorescence intensities for the sample. Fluorescence intensities were extracted on an arbitrary unit scale of 0 (no fluorescence signal) to 255 (saturated fluorescence signal). Fluorescence intensities were averaged across peripheral and core regions, determined through averaging pixel intensities within regions of the cross‐sectional area. Peripheral regions were defined as 5% of the total length of the measured radius, taken from the edges of the spheroid from either side of the measured lines to account for 10% of the total line length. Core regions were defined as 10% of the total length of the measured radius, taken from the core of the spheroid. The nuclear and cytosolic YAP intensities were determined in ImageJ. A mask was generated by thresholding the nuclei staining and the YAP intensity was determined in regions within and outside the masked region‐of‐interest to yield the nuclear‐to‐cytosolic YAP ratio.

### Adjusting Duration of Spheroid Confinement by Hydrogel Cups

Spheroids were generated within the cup cavities as outlined in Section 2.4. Cups were enzymatically digested after 5, 7, and 10 days of culture by incubation of confined spheroids in Hank's buffer supplemented with 1 mg mL^−1^ (10 U mL^−1^) alginate lyase at physiological conditions. Thereafter, spheroids were manually transferred using a sterile 1 mL pipette tip to a low‐adherent 96‐well U‐bottom plate and kept in culture for a total culture time of 10 days. We opted for the U‐bottom plates to grow the spheroids post‐confinement to match the culture conditions of the unconfined spheroids (see also “Unconfined and confined spheroid generation” in the Experimental Section).

### Drug‐Mediated Inhibition of Mechanotransduction

Confined spheroids were cultured for 5 days as outlined prior. After 5 days of culture, the media was replaced with fresh media supplemented with drugs influencing mechanotransduction pathways. Media was supplemented with a complexation inhibitor of the YAP with TEAD protein (peptide 17; 100 nm),^[^ ^75^, ^76^ ^]^ a selective inhibitor of Rho‐associated, coiled‐coil containing protein kinase (Y‐27632; 10 µm),^[^ ^75^, ^77^ ^]^ or a selective inhibitor of myosin II (blebbistatin; 50 µm).^[^ ^77^ ^]^ Samples were exposed to these drugs for 48 h prior to being fixed in 4% w/v formaldehyde for 48 h and washed in PBS for further downstream analysis.

### Statistical Analysis

Unless stated otherwise, all samples were prepared in triplicates (*n* = 3) within individual experiments and each experiment was repeated three times (N = 3) to study variability within and between experiments. Raw data were expressed as mean ± standard deviation. Statistical analyses between experimental groups were conducted using a one‐way ANOVA with the Tukey post‐hoc test. Statistical analyses investigating time points within the same group were conducted an independent sample t‐test. Statistically significant differences between experimental groups of interest were depicted as ^*^(*p* <0.05), ^**^(*p* <0.01), ^***^(*p* <0.001), or ^****^(*p* <0.0001), and non‐significance was indicated where appropriate with *ns*. Statistical analyses were made using GraphPad Prism software, version 10.

## Conflict of Interest

The authors declare no conflict of interest.

## Author Contributions

B.G.S., K.A.K., and J.J.G. conceived the ideas and designed the experiments. B.G.S., P.T., and J.C. conducted the experiments and analyzed the data. All authors interpreted the data and contributed to writing the manuscript.

## Supporting information



Supporting Information

Supplemental Video 1

Supplemental Video 2

Supplemental Video 3

Supplemental Video 4

## Data Availability

The data that support the findings of this study are available from the corresponding author upon reasonable request.
