# Supplementary material for: Interfacial Stress Regulates Plasticity and Drug Resistance at the Breast Cancer‐Host Interface
Source: Adv Sci (Weinh). 2025 Jun 26;12(36):e09361. doi: 10.1002/advs.202509361 (PMC12462989; doi:10.1002/advs.202509361)
Supplement: Supplementary file 1 — Supporting Information [file ADVS-12-e09361-s001.docx]

# Supporting Information

**Interfacial Stress Regulates Plasticity and Drug Resistance at the Breast Cancer-Host Interface**

*B.G. Soliman, P. Tian, J. Cui, K. A. Kilian, J. J. Gooding*

**Extended Experimental Section**

*Viability and metabolic activity:* Cell viability was determined after one and five days of culture. Unconfined and confined spheroids were washed in PBS and then incubated in PBS supplemented with 1 *μ*g ml^−1^ calcein-AM and 1 *μ*g mL^−1^ propidium iodide for 30 minutes. Samples were then washed once with PBS and imaged on a Zeiss LSM 800 confocal microscope coupled with Zen Blue software. Images were processed using ImageJ. Cell viability was determined as the percentage of live cells as compared to the total cell number, being the sum of live and dead cells. Metabolic activity was determined at day 1, 3, 5 and 7 through Alamar blue® metabolic activity assay according to the manufacturer’s protocol. Briefly, fresh media was supplemented with Alamar blue® reagent at a 1:10 ratio and added onto the unconfined and confined spheroids. Samples were then incubated for 2 hours at physiological conditions (37 °C, 5% CO_2_). The Alamar blue® reagent-supplemented media was then collected and fluorescence signal observed from this media was measured (λ_ex_ = 530 nm, λ_em_ = 590 nm) using a ClarioStar Plus microplate reader. Metabolic activity was directly correlated to the measured fluorescence intensity after normalization to Alamar blue® reagent-supplemented media that had not been in contact with cells.

*Drop-on-demand printed ink droplet visualization and quantification:* A custom-made visualization system was utilized wherein a high-speed camera captured droplets dispensed from the drop-on-demand printer nozzle. Built-in software analyzed captured images to determine the droplet volume based on the assumption that the dispended droplets were spherical. Printing pressure was systematically varied to establish the relationship between printing pressure and the volume of the resulting droplet.

*Drop-on-demand printed hydrogel cup cavity height determination:* To determine the height of the cup cavity, hydrogel cups were printed onto coverslips and transferred on a Physica MCR301 rheometer (Anton Paar, Germany), outfitted with an optical Peltier plate setup and plate-plate geometry (25 mm diameter). The plate geometry was set above the hydrogel cups and lowered at a constant speed of 10 μm s^-1^, continuously registering force. The force remained constant until contact was made with the top of the hydrogel, at which point a sharp increase in force was observed. The hydrogel cup height was defined as the largest distance between both plates of the plate-plate geometry at which the force increase reached a force change of 0.5 N mm^-1^ distance.

*Custom-made ImageJ macro for evaluation of immunofluorescent confocal images:* Confocal immunofluorescent images of unconfined and confined samples (see ‘Immunofluorescent Staining, Imaging and Quantification’ in the Experimental Section) were evaluated through the following custom-made ImageJ macro:

dir = getDirectory( "Choose the Directory" );

list = getFileList( dir );

outputDir = dir;

splitDir= dir + "/Layer ROIs/";

File.makeDirectory(splitDir);

for ( i=0; i<1; i++ ) {

open( dir + list[0] ); // Opens File

run("8-bit");

roiManager("reset");

run("Split Channels");

close();

close();

title = getTitle();

run("Clear Results");

setTool(4);

waitForUser("set line 1 and press OK");

roiManager("Add");

run("Measure");

waitForUser("set line 2 and press OK");

roiManager("Add");

run("Measure");

waitForUser("set line 3 and press OK");

roiManager("Add");

roiManager("Save", splitDir+title+"Roi.zip");

run("Measure");

run("Read and Write Excel", "sheet=Line_widths file=["+outputDir+"raw data"+".xlsx]");

run("Clear Results");

close();

close();

}

for ( i=0; i<list.length; i++ ) {

open( dir + list[i] ); // Opens File

run("8-bit");

run("Split Channels");

close();

close();

title = getTitle();

roiManager("select", 0);

profile = getProfile();

for (j=0; j<profile.length; j++)

setResult("Value", j, profile[j]);

updateResults();

run("Read and Write Excel", "sheet=Channel_555 file=["+outputDir+"raw data"+".xlsx]");

run("Clear Results");

roiManager("select", 1);

profile = getProfile();

for (j=0; j<profile.length; j++)

setResult("Value", j, profile[j]);

updateResults();

run("Read and Write Excel", "sheet=Channel_555 file=["+outputDir+"raw data"+".xlsx]");

run("Clear Results");

roiManager("select", 2);

profile = getProfile();

for (j=0; j<profile.length; j++)

setResult("Value", j, profile[j]);

updateResults();

run("Read and Write Excel", "sheet=Channel_555 file=["+outputDir+"raw data"+".xlsx]");

run("Clear Results");

close();

title = getTitle();

roiManager("select", 0);

profile = getProfile();

for (j=0; j<profile.length; j++)

setResult("Value", j, profile[j]);

updateResults();

run("Read and Write Excel", "sheet=Channel_647 file=["+outputDir+"raw data"+".xlsx]");

run("Clear Results");

roiManager("select", 1);

profile = getProfile();

for (j=0; j<profile.length; j++)

setResult("Value", j, profile[j]);

updateResults();

run("Read and Write Excel", "sheet=Channel_647 file=["+outputDir+"raw data"+".xlsx]");

run("Clear Results");

roiManager("select", 2);

profile = getProfile();

for (j=0; j<profile.length; j++)

setResult("Value", j, profile[j]);

updateResults();

run("Read and Write Excel", "sheet=Channel_647 file=["+outputDir+"raw data"+".xlsx]");

run("Clear Results");

close();

}

for (a=0;a<nImages;a++) {

selectImage(a+1);

title = getTitle;

print(title);

//ids[i]=getImageID;

}

run("Close All");

**Supporting Figures**


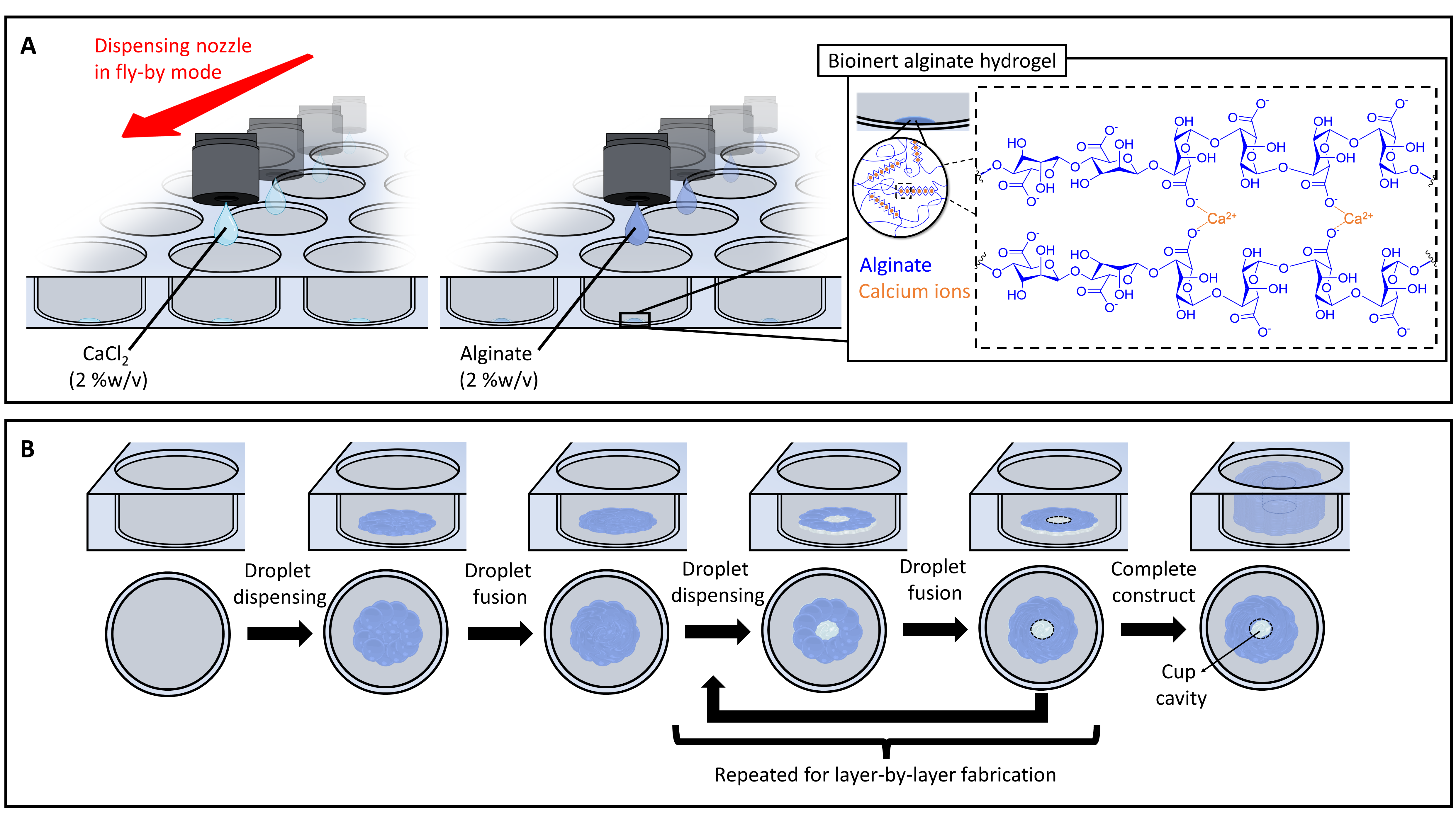


**Figure S1. Drop-on-demand printing of cup-shaped alginate hydrogels.** (**A**) Schematic representation of drop-on-demand printing, wherein a printhead “flies” over wells of a 96 well plate, rapidly dispensing sequential droplets of calcium chloride and alginate to print alginate hydrogels in high throughput. (**B**) Side- and top-view of the droplet-by-droplet, layer-by-layer, fabrication process of cup-shaped alginate hydrogels. Alginate hydrogels are dispensed across the base of the well plate to form a flat alginate slab upon droplet fusion. Thereafter, alginate hydrogel walls are dispensed on top of this slab that similarly merge to form an alginate hydrogel ring to complete the cup structure, leaving a circular cavity in the center. Figure contains elements created with BioRender.com.


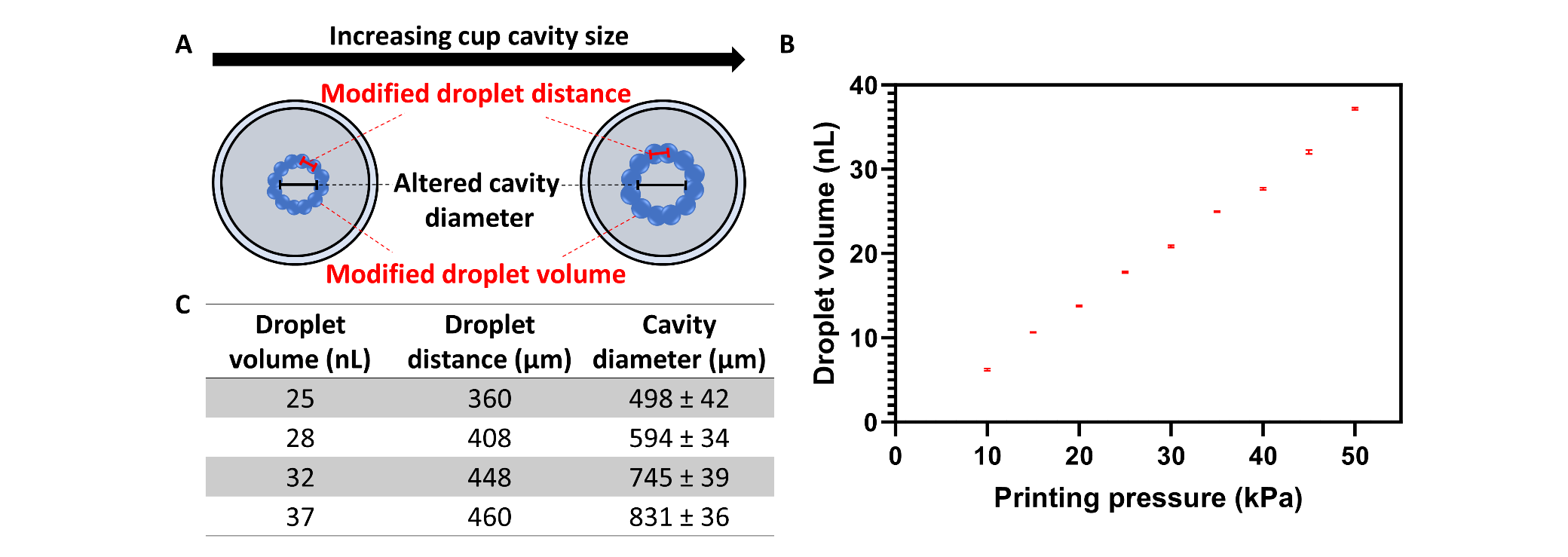


**Figure S2. Controlling the cavity size of the alginate-based cup model by adjusting drop-on-demand printing parameters.** (**A**) Printing pressure was adjusted to modify the volume of individual ink droplets. The droplet distance was modified in the printing code to maintain the circular shape of the cavity. Increasing both droplet volume and droplet distance allowed altering of the cavity diameter. (**B**) The relationship between droplet volume and printing pressure. (**C**) The relationship between droplet volume (enabling altering of the printing pressure), droplet distance and the diameter of the resulting cup cavity. spheroid size was measured from Brightfield images taken after seven days of culture. Graphs show mean ± SD. N = 3. Figure contains elements created with BioRender.com.


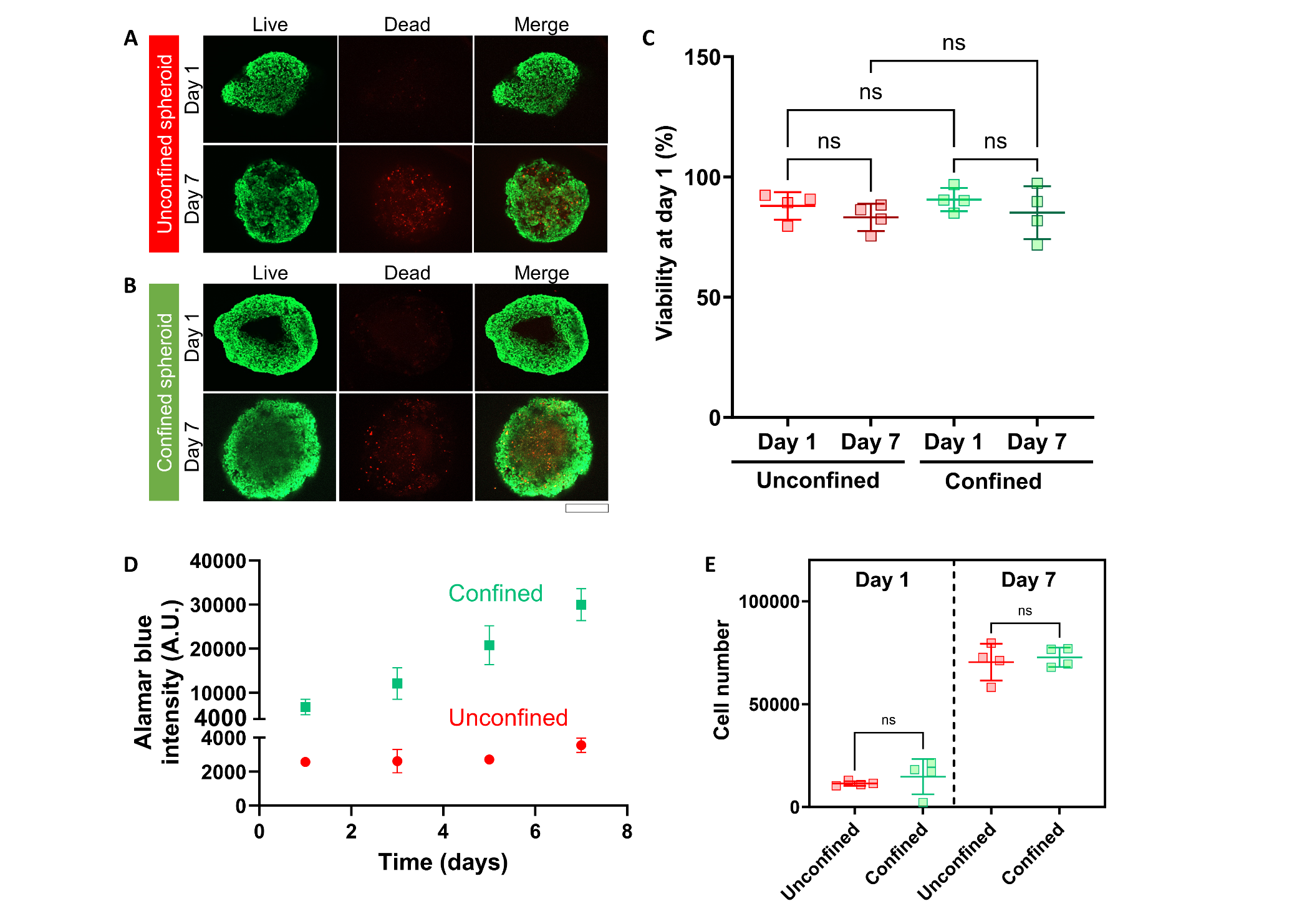


**Figure S3. Cytocompatibility of the drop-on-demand printing process.** (**A** to **B**) Representative images of Live/Dead staining after 1 (A) and 5 (B) days of culture, with live cells in green and dead cells in red. (**C**) Cell viability was calculated from Live/Dead images and deﬁned as the number of live cells divided by the total number of cells. Independent sample t-test between experimental groups. (**D**) Metabolic activity of unconfined and confined spheroids. (**E**) Average cell number per unconfined and confined spheroids after 1 and 7 days of culture. One-way ANOVA with Tukey post-hoc test to compare multiple experimental groups. Statistical non-significance was indicated with ns (= not significant). Graph shows mean ± SD. N = 3.


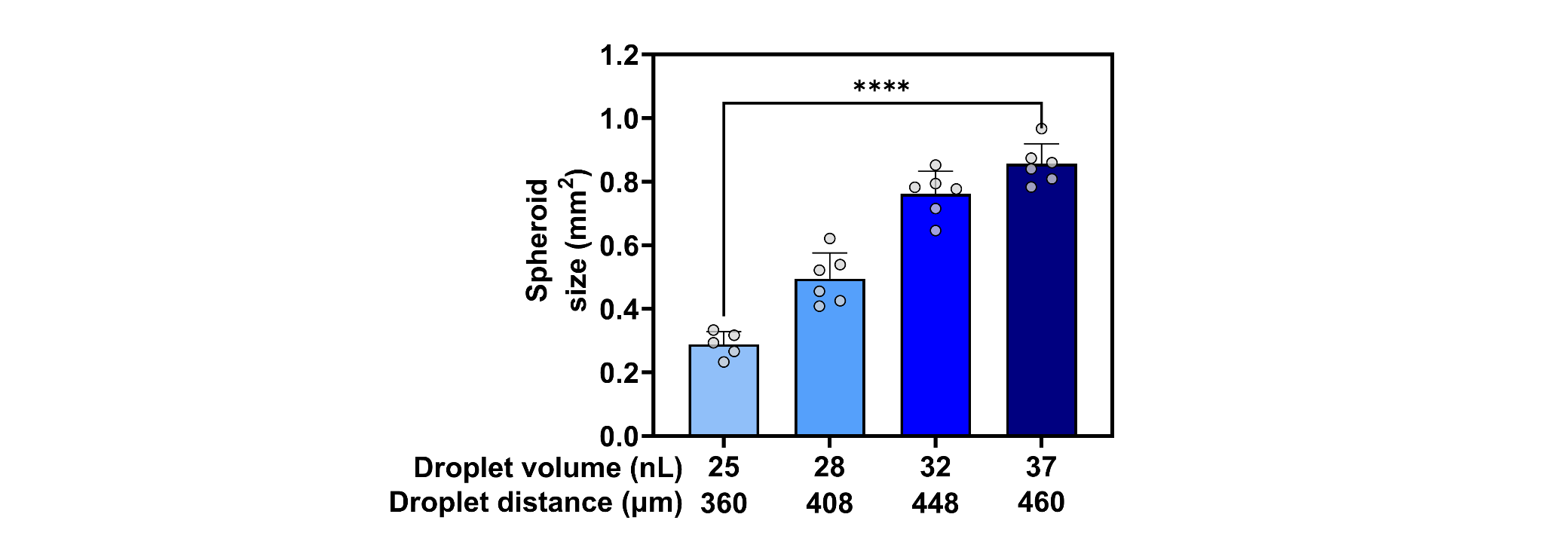


**Figure S4. Controlling spheroid size by adjusting the cavity design of the cup hydrogel model.** MCF7 spheroid size was measured from Brightfield images taken after seven days of culture. One-way ANOVA with Tukey post-hoc test to compare multiple experimental groups. Statistical differences are depicted with **** (0.001 < p). Graphs show mean ± SD. N = 3.


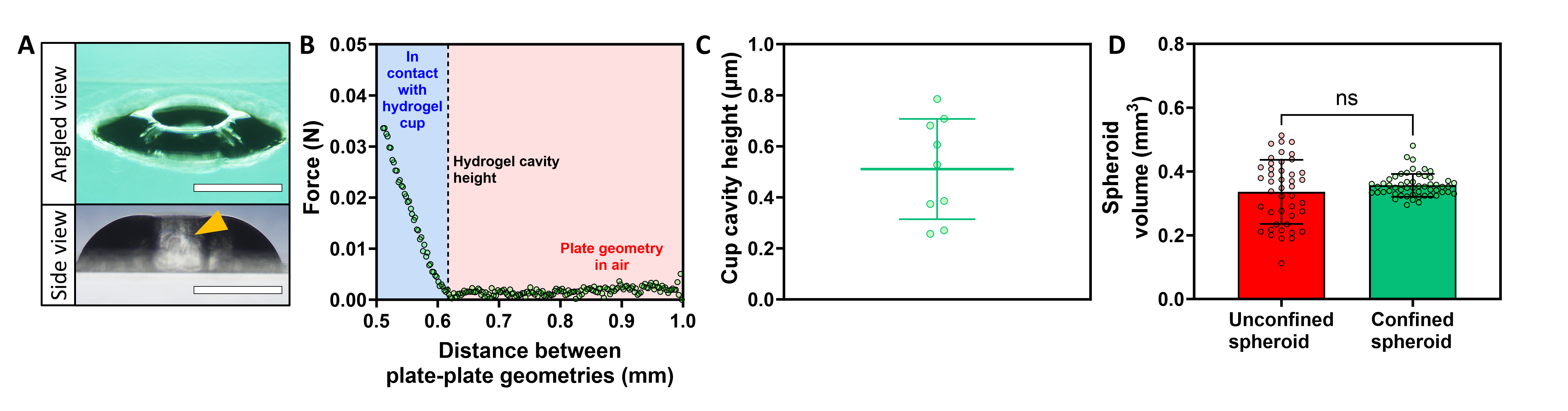


**Figure S5. Quantification of hydrogel cup cavity and spheroid volume.** (**A**) Representative images of the printed hydrogel cup. The yellow arrow indicates the presence of the cylindrical cavity in the side view. (**B**) Representative dataset for hydrogel cup height determination as quantified by a force change that was registered upon a Peltier plate setup with plate-plate geometry contacting the top of the hydrogel. (**C**) The quantified hydrogel cup height was averaged across all samples. (**D**) Spheroid volume was calculated and compared to the volume of unconfined spheroids as derived from Brightfield imaging. Independent sample t-test between experimental groups. Statistical non-significance was indicated with ns (= not significant). Scale bar = 1 mm. Graphs show mean ± SD. N = 9.


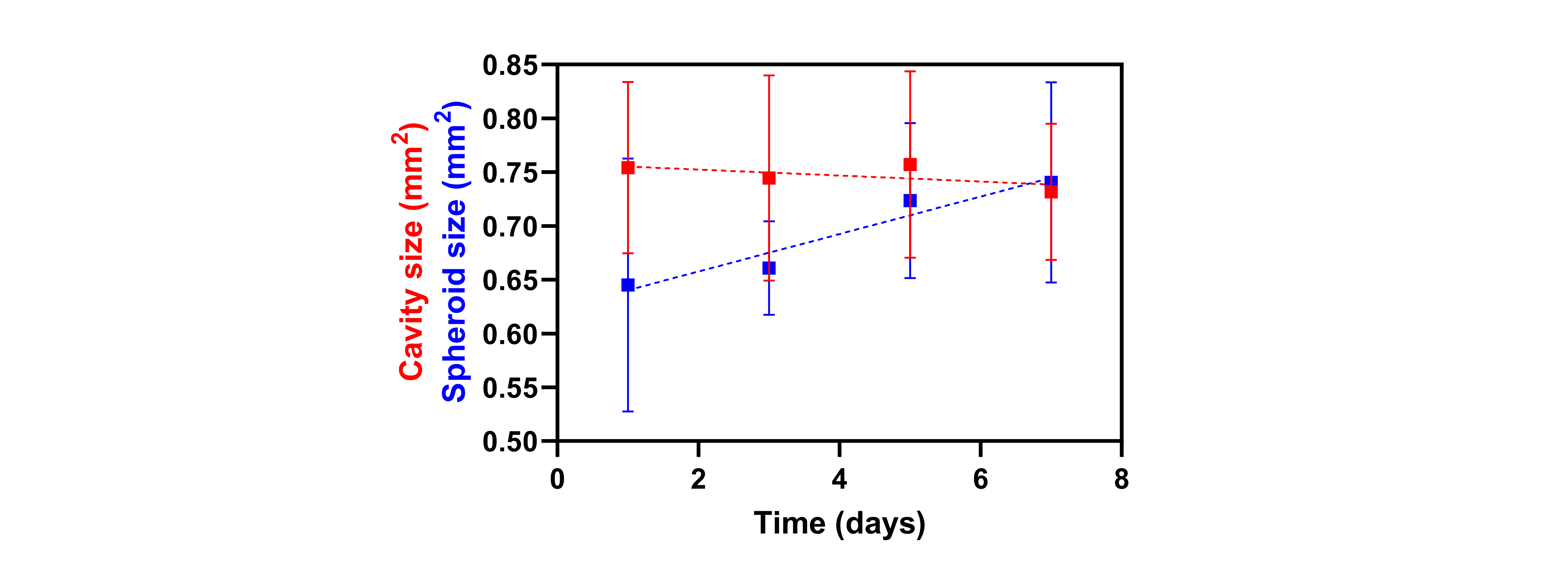


**Figure S6. Spheroid growth tracking in confined conditions.** Cavity size (red) and spheroid size (blue) was quantified from Brightfield images at various time points during confined spheroid culture. Graphs show mean ± SD. N = 36.


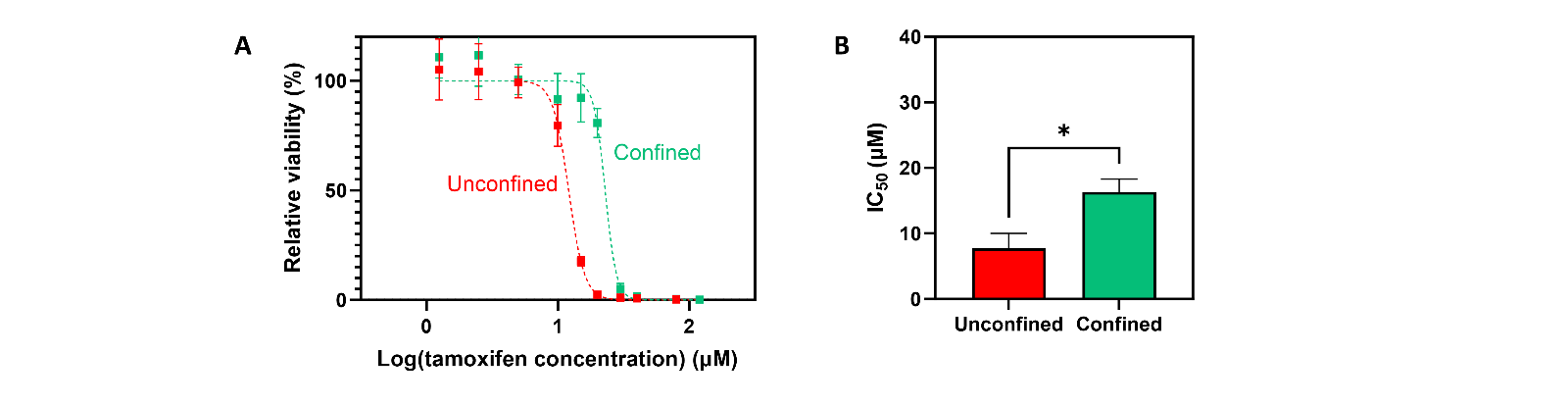


**Figure S7. Sensitivity of unconfined and confined spheroids to cytotoxic drug tamoxifen.** (**A**) Dose-response curves demonstrating doxorubicin-dose dependent MCF-7 cell survival after five days of drug interrogation in unconfined and confined spheroids. Spheroids were cultured in media without any doxorubicin as a 100% viability control to which the measured viability in the drug-exposed samples could be normalized. Spheroids were cultured in media with 30 %w/v ethanol as a positive control for cell death to establish background signal at 0% viability. Trendlines were derived from a logarithmic variable slope non-linear regression curve fitting. (**B**) Half-maximal inhibitory concentration (IC_50_) was extrapolated from trendlines across three experimental repeats and averaged to compare drug sensitivity in unconfined and confined spheroids. Independent sample t-test between experimental groups. Statistical differences are depicted with * (0.05 < p). Graphs show mean ± SD. N = 3.


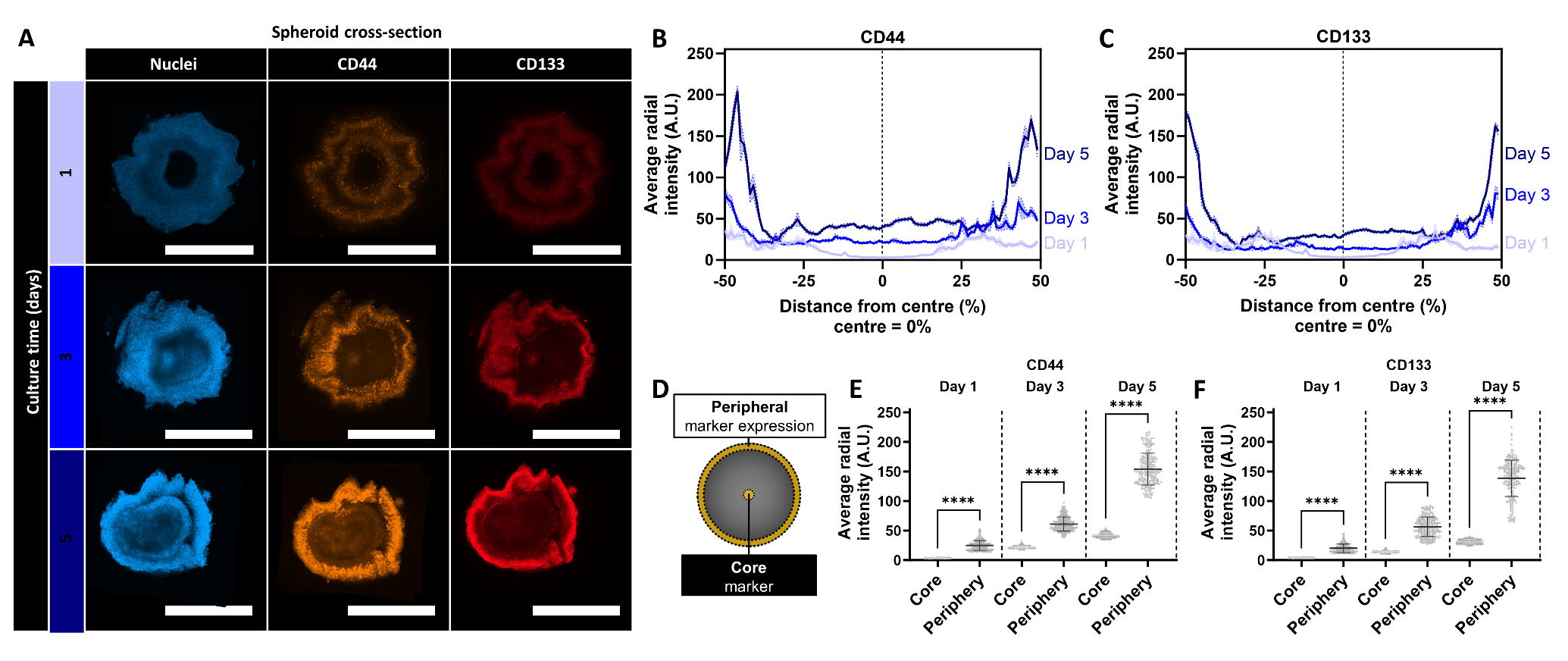


**Figure S8. The emergence of cellular heterogeneity at hydrogel-spheroid interface during early stages of spheroid growth. A**) Representative images of immunofluorescence staining of CD44 (orange) and CD133 (red) in unconfined and confined spheroids. Images are maximum intensity projections of the slices at the median of spheroids. (**B** to **C**) Quantification of spatial heterogeneity in CD44 (B) and CD133 (C) expression as quantified through the relative fluorescence intensity from immunofluorescence-stained images. The fluorescence intensity was quantified across the cross-sectional area and expressed as the average radial fluorescence intensity defined as the fluorescence intensity at a given percentual distance from the peripheries of the spheroid towards the spheroid core. (**D** to **F**) Fluorescence intensities were compared between peripheral and core regions of confined spheroids (D), defined as the outside 5% sections and inside 10% sections of the total diameter, respectively. Differential expression between core and periphery regions were visualized for CD44 (E) and CD133 (F). Each individual point represents the pixel fluorescent intensity at the set distance within the cross-sectional area, averaged across the analyzed spheroids. Independent sample t-test between experimental groups. Statistical differences are depicted with **** (0.0001 < p). Scale bars = 1 mm. Graphs show mean ± SD. N = 3.


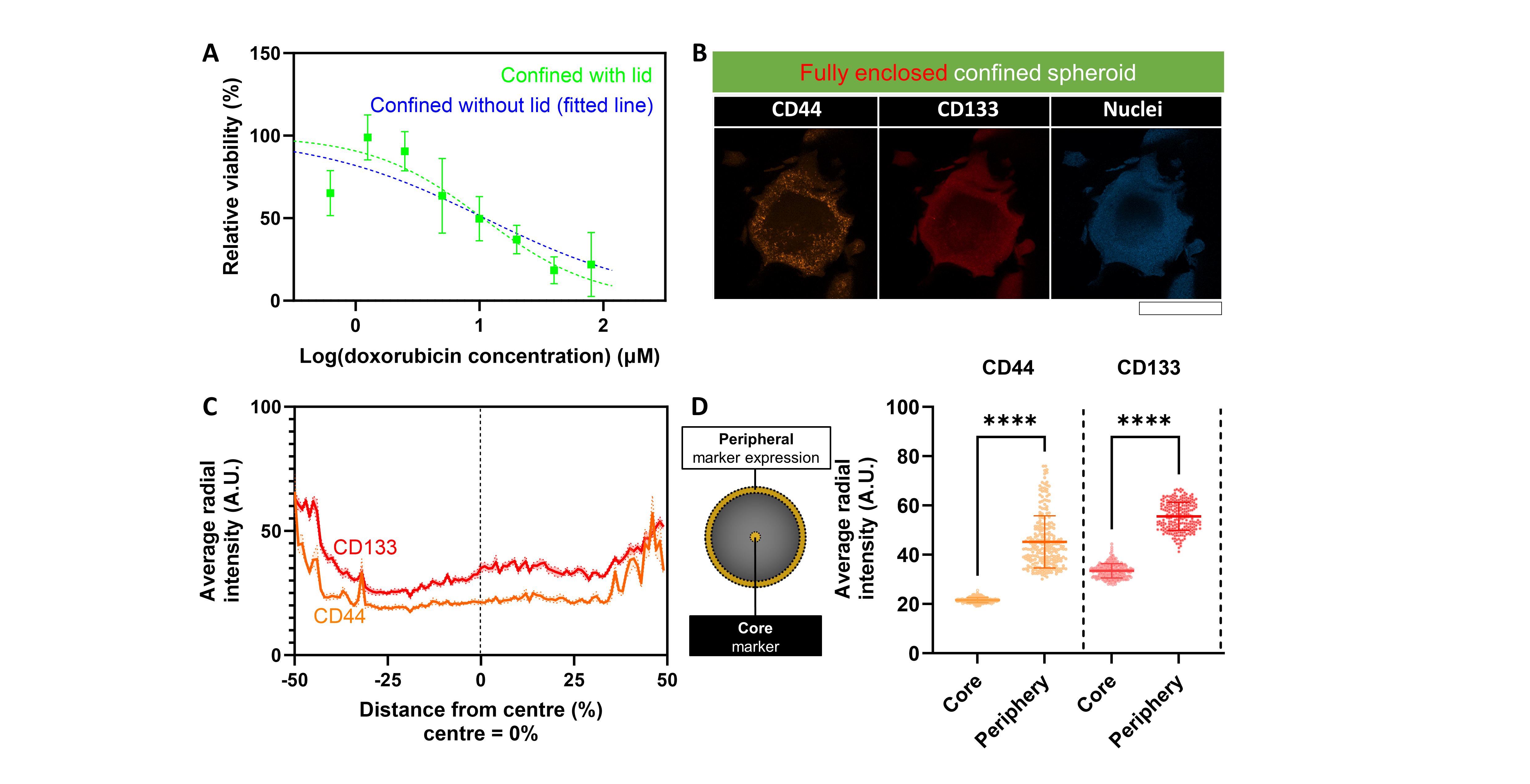


**Figure S9. Axial confinement of spheroids was unnecessary to affect confinement-induced drug insensitivity and the emergence of a drug-resistant peripheral phenotype.** (**A**) Dose-response curve for axially confined spheroids**.** Axially confined spheroids were prepared similarly to the confined spheroids using drop-on-demand printing. Post cell dispensing, a lid was fabricated on top of the hydrogel cup to affect axial as well as lateral confinement. A trendline (blue) for laterally confined spheroids (i.e. without the lid) was included for comparison. (**B**) Representative images of immunofluorescence staining of CD44 (orange) and CD133 (red). Representative images were displayed as maximum intensity projections of the slices at the median of spheroids. (**C** to **D**) Quantification of spatial heterogeneity in CD44 (C) and CD133 (D) expression. Fluorescence intensities were determined in the spheroid’s peripheral regions, defined as the outside 5% sections of the total diameter, respectively. Each individual point represents the pixel fluorescent intensity at the set distance within the cross-sectional area, averaged across the analyzed spheroids. One-way ANOVA with Tukey post-hoc test to compare multiple experimental groups. Independent sample t-test between experimental groups. Statistical differences are depicted with * (0.05 < p) and **** (0.001 < p). Scale bar = 1 mm. Graphs show mean ± SD. N = 3.


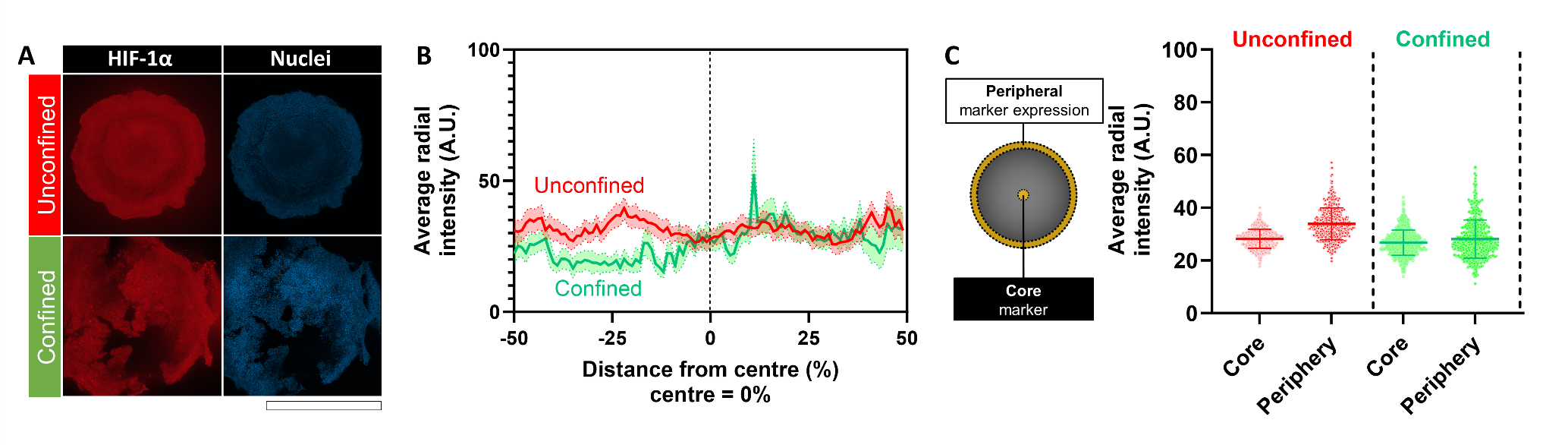


**Figure S10. Hypoxia in unconfined and confined spheroids.** (**A**) Representative images of immunofluorescence staining of HIF-1α (red) in unconfined and confined spheroids. Images are maximum intensity projections of the slices at the median of spheroids. (**B**) Quantification of spatial heterogeneity in HIF-1α expression as quantified through the relative fluorescence intensity from immunofluorescence-stained images. The fluorescence intensity was quantified across the cross-sectional area and expressed as the average radial fluorescence intensity defined as the fluorescence intensity at a given percentual distance from the peripheries of the spheroid towards the spheroid core. (**C**) HIF-1α fluorescence intensities were compared between peripheral and core regions of confined spheroids (D), defined as the outside 5% sections and inside 10% sections of the total diameter, respectively. Independent sample t-test between experimental groups. Statistical non-significance was indicated with ns (= not significant). Scale bars = 1 mm. Graphs show mean ± SD. N = 3.


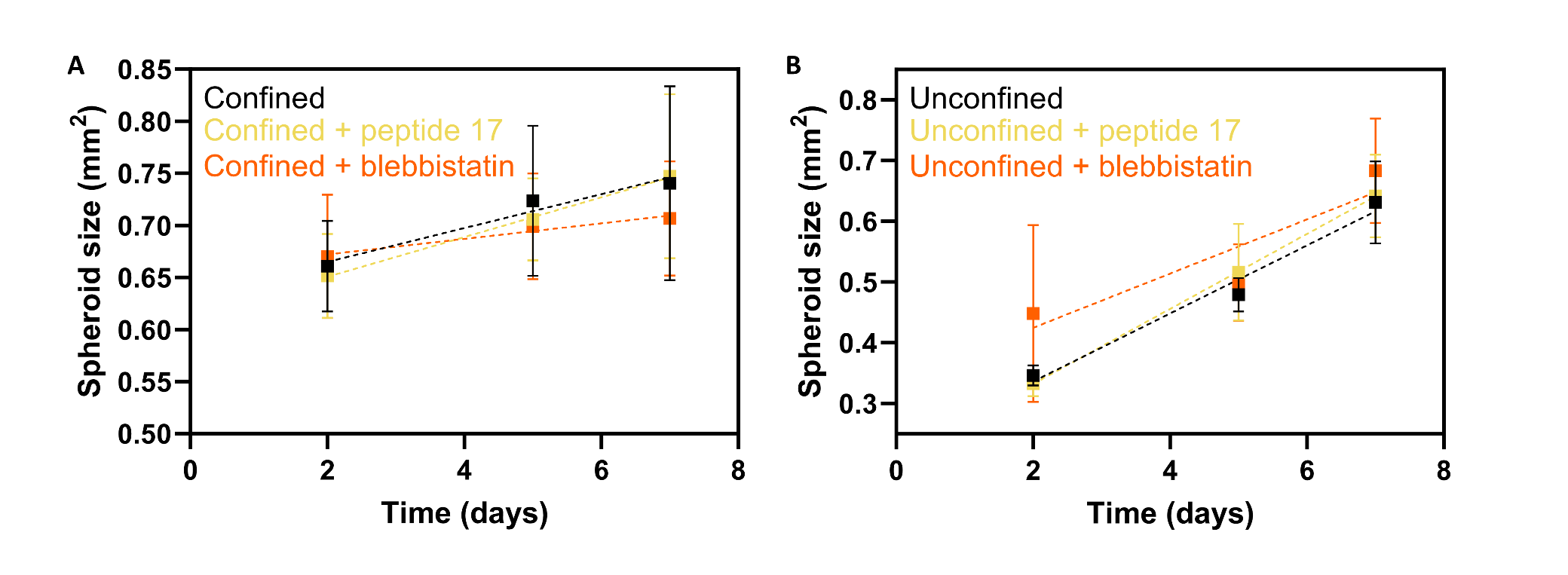


**Figure S11. Spheroid growth tracking in spheroids treated with pharmaceutical inhibitors of the mechanotransduction pathway.** Spheroid size was quantified from Brightfield images at various time points during confined spheroid culture (**A**) and unconfined spheroid culture (**B**). Graphs show mean ± SD. N = 18.

**Supporting Videos**

Supporting Videos are available as separate media files. Captions for the Supporting Videos are depicted below.

**Supporting Video 1. 3D rendered heatmap of CD44 expression in an unconfined spheroid.** Scale bar = 100 μm.

**Supporting Video 2. 3D rendered heatmap of CD44 expression in a confined spheroid.** Scale bar = 100 μm.

**Supporting Video 3. Animated slices of Z-stack taken from an unconfined spheroid.** Individual channels show nuclei (blue), CD44 (orange) and CD133 (red). Scale bar = 100 μm.

**Supporting Video 4. Animated slices of Z-stack taken from a confined spheroid.** Individual channels show nuclei (blue), CD44 (orange) and CD133 (red). Scale bar = 100 μm.
